# Marine-derived fungi as biocatalysts

**DOI:** 10.3389/fmicb.2023.1125639

**Published:** 2023-02-27

**Authors:** Jorge R. Virués-Segovia, Salvador Muñoz-Mira, Rosa Durán-Patrón, Josefina Aleu

**Affiliations:** Departamento de Química Orgánica, Facultad de Ciencias, Campus Universitario Río San Pedro s/n, Torre sur, 4ª Planta, Universidad de Cádiz, Cádiz, Spain

**Keywords:** marine fungi, biocatalyst, biotransformation, biodegradation, bioremediation

## Abstract

Marine microorganisms account for over 90% of ocean biomass and their diversity is believed to be the result of their ability to adapt to extreme conditions of the marine environment. Biotransformations are used to produce a wide range of high-added value materials, and marine-derived fungi have proven to be a source of new enzymes, even for activities not previously discovered. This review focuses on biotransformations by fungi from marine environments, including bioremediation, from the standpoint of the chemical structure of the substrate, and covers up to September 2022.

## 1. Introduction

The marine ecosystem covers over 70% of the earth’s surface and its rich biodiversity is mostly yet to be discovered. Because of its unique physical and chemical conditions, the marine environment offers rich microbial diversity (bacteria, fungi, algae, plankton, and viruses) characterized by metabolic pathways and enzymatic systems different from those of their terrestrial counterparts. That is why marine microorganisms have proved to be interesting and are a potential source of structurally unique and biologically active natural products (Jensen and Fenical, 1996; Saleem et al., 2007; Jin et al., 2016). For example, the sesquiterpenes chloriolins A, B, and C, isolated from an unidentified marine fungus, chlorinated analogs of the terrestrial fungal coriolins (Cheng et al., 1994), or dendryphiellin A obtained from the obligate marine deuteromycete *Dendryphiella salina*, with an unprecedented structure (Guerriero et al., 1988), demonstrate the potential of marine fungi as a source of unique chemistry.

Marine-derived microorganisms are naturally adapted to extreme temperature, acidity, pressure and/or salt concentration in the ocean; therefore, they can be an important source of new enzymes with interesting characteristics, i.e., high salt tolerance, hyperthermostability, barophilicity and cold adaptivity. These ecological features of habitat in which they thrive impact on their metabolic functions enabling (Rocha et al., 2012c). For example, the esterase isolated from the marine yeast *Yarrowia lipolytica* CL180 showed psychrophilic activity and, in fact, still showed 40% of the maximum activity at 10°C, making it a very attractive enzyme for potential application in the production of a thermolabile chemical (Trincone, 2011).

The occurrence of marine fungi has been reported in different substrates as sponges, algae, wood, tunicates, sediments, mollusks, corals, plants, fish… (Bonugli-Santos et al., 2015; Grossart et al., 2019). Interestingly, the holobiont environment has proved to be an abundant reservoir of bioactive compounds, affecting the marine host and their microbiome (Yarden, 2014).

In recent decades, biocatalysis has become an increasingly valuable tool for the chemical synthesis of novel drug derivatives, agrochemicals and fragrances with improved properties, or precursor/intermediate molecules involved in production processes owing to the capability of biological systems to conduct regio- and stereoselective chemical reactions that cannot be performed by traditional synthetic methods. Biocatalysis is also an interesting alternative in the context of Green Chemistry where gentle, and usually cheaper reaction conditions are employed (water as the reaction medium, physiological pH and temperature…; Muñoz Solano et al., 2012). Moreover, in recent years, microorganisms that mimic mammalian metabolism have been employed for the pharmacological and toxicological evaluation of bioactive compounds (Kuban et al., 2010).

Over the last few decades, marine mycology has started to become an incipient source of new natural products as potential lead compounds. Up to 2021, 1901 species were described and more than 3500 secondary metabolites isolated (Jin et al., 2016; Gonçalves et al., 2022). Despite their widespread distribution and abundance, marine-derived fungi, which are generally known as fungi isolated from the marine environment (Pang et al., 2016), remain an underexplored and promising reservoir of enzymes that can potentially be used for biotransformation and biodegradation purposes (Birolli et al., 2019; Gonçalves et al., 2022).

This review focuses on the biotransformation of natural and synthetic compounds by marine-derived fungi with the aim of providing some interesting insights into their enzymatic systems and possible application in chemical transformations as part of synthetic procedures in drug design or other products of industrial interest. A description of the potential use of these fungi as an alternative bioprocess for the degradation of pollutants is also given. The review covers articles published until September 2022.

## 2. Biotransformation of natural products

### 2.1. Terpenes

Terpenoids are natural compounds which are essential for living organisms for growth, development and defense against threats. Due to their wide array of bioactivity, they are widely used in agrochemicals, drugs, fragrances, flavoring and pigments (Gozari et al., 2021).

Monoterpenes are an important component of essential oils and mainly play an ecological role in plants. In view of the wide variety of applications of these compounds, many attempts have been made to modify their structure to enhance their properties.

**Figure 1:**
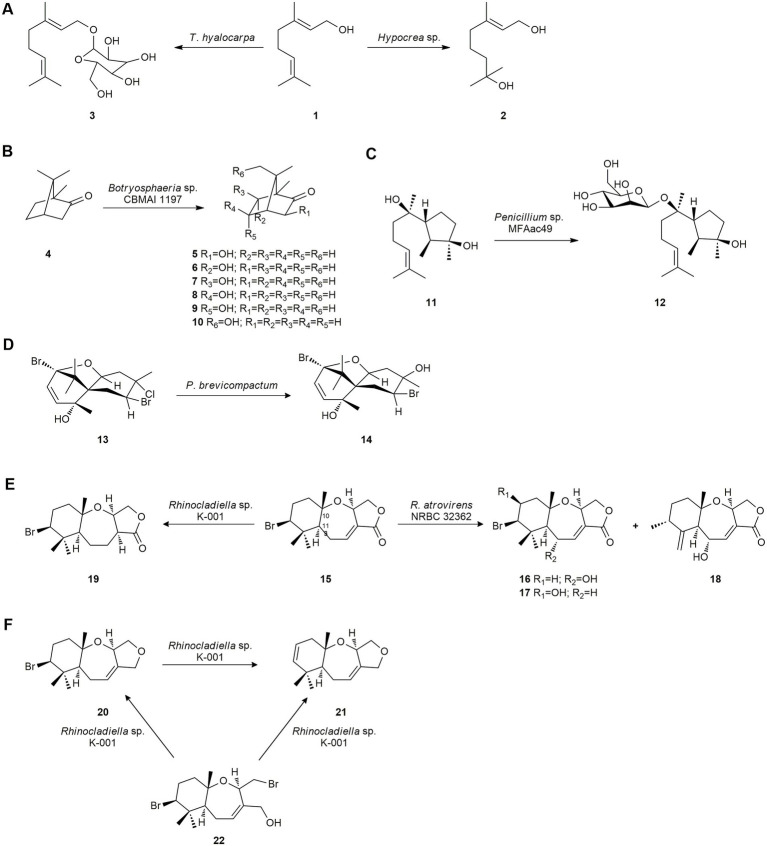
Terpene biotransformations. **(A)** Geraniol (**1**) biotransformation by *Thielavia hyalocarpa* and *Hypocrea* sp. (Yun et al., 2015). **(B)** Camphor (**4**) biotransformation by *Botryosphaeria* sp. (de Jesus et al., 2017). **(C)** Cyclonerodiol (**11**) mannosidation by the marine-derived fungus *Penicillium* sp. MFAac49 (Li et al., 2007). **(D)** Pacifenol (**13**) bioconversion by *Penicillium brevicompactum* (San-Martín et al., 2008). **(E)** Biotransformation of aplysistatin (**15**) by *Rhinocladiella* sp. K-001 and *Rhinocladiella atrovirens* NRBC 32362. **(F)** Biotransformation of hydroxypalisadin B (**22**) and palisadin A (**20**) by *Rhinocladiella* sp. K-001 (Koshimura et al., 2009).

Leutou et al. (2009) studied the biotransformation of the flavor monoterpene geraniol (**1**) using several microorganisms. Only the fungus *Hypocrea* sp., isolated from the marine alga *Undaria pinnatifida* found in South Korea, was able to hydroxylate **1** yielding 1,7-dihydroxy-3,7-dimethyl-(*E*)-oct-2-ene (**2**). Subsequently, these authors obtained the monoterpene glycoside 1-*O*-(*α*-d-mannopyranosyl) geraniol (**3**) by using the marine-derived fungus *Thielavia hyalocarpa* from geraniol (**1**; Figure 1A). This fungus was isolated from a mudflat collected at Suncheon Bay, Korea (Yun et al., 2015).

Camphor (**4**) is a major constituent of the essence of *Salvia officinalis* and is used commercially as a moth repellent and component of pharmaceutical products. The fungus *Botryosphaeria* sp. CBMAI 1197, isolated from the red marine alga *Bostrychia radicans* collected in the South Atlantic Ocean off the northern coast of São Paulo, Brazil, was used to biotransform this compound, affording the following compounds: 3-*exo*-hydroxycamphor (**5**), 6-*endo*-hydroxycamphor (**6**), 6-*exo*-hydroxycamphor (**7**), 5-*exo*-hydroxycamphor (**8**), 5-*endo*-hydroxycamphor (**9**) and 8-hydroxycamphor (**10**; Figure 1B). These monohydroxylations are difficult to perform using conventional methods (de Jesus et al., 2017).

The bioactive sesquiterpene cyclonerodiol (**11**), a plant growth regulatory active constituent, was biotransformed by the marine-derived fungus *Penicillium* sp. (MFAac49), isolated from the brown alga *Sargassum thunbergii* collected at Songjeung Beach in Busan, Korea. This fungus produced a mannosidation obtaining cyclonerodiol mannopyranoside **12** as a biotransformation product with moderate yield (Figure 1C). Microbial mannosidation is a very rare reaction, which was produced in terpenes by marine fungi for the first time (Li et al., 2007).

Later, San-Martín et al. (2008) described the microbial transformation of pacifenol (**13**), a bioactive polyhalogenated sesquiterpene with anti-microbial activity, by the facultative halotolerant fungus *Penicillium brevicompactum,* isolated from the sponge *Cliona* sp. Hydroxylated compound **14** was obtained (Figure 1D) and studies showed that it had moderate anti-bacterial activity against *Staphylococcus enteriditis*.

**Figure 2:**
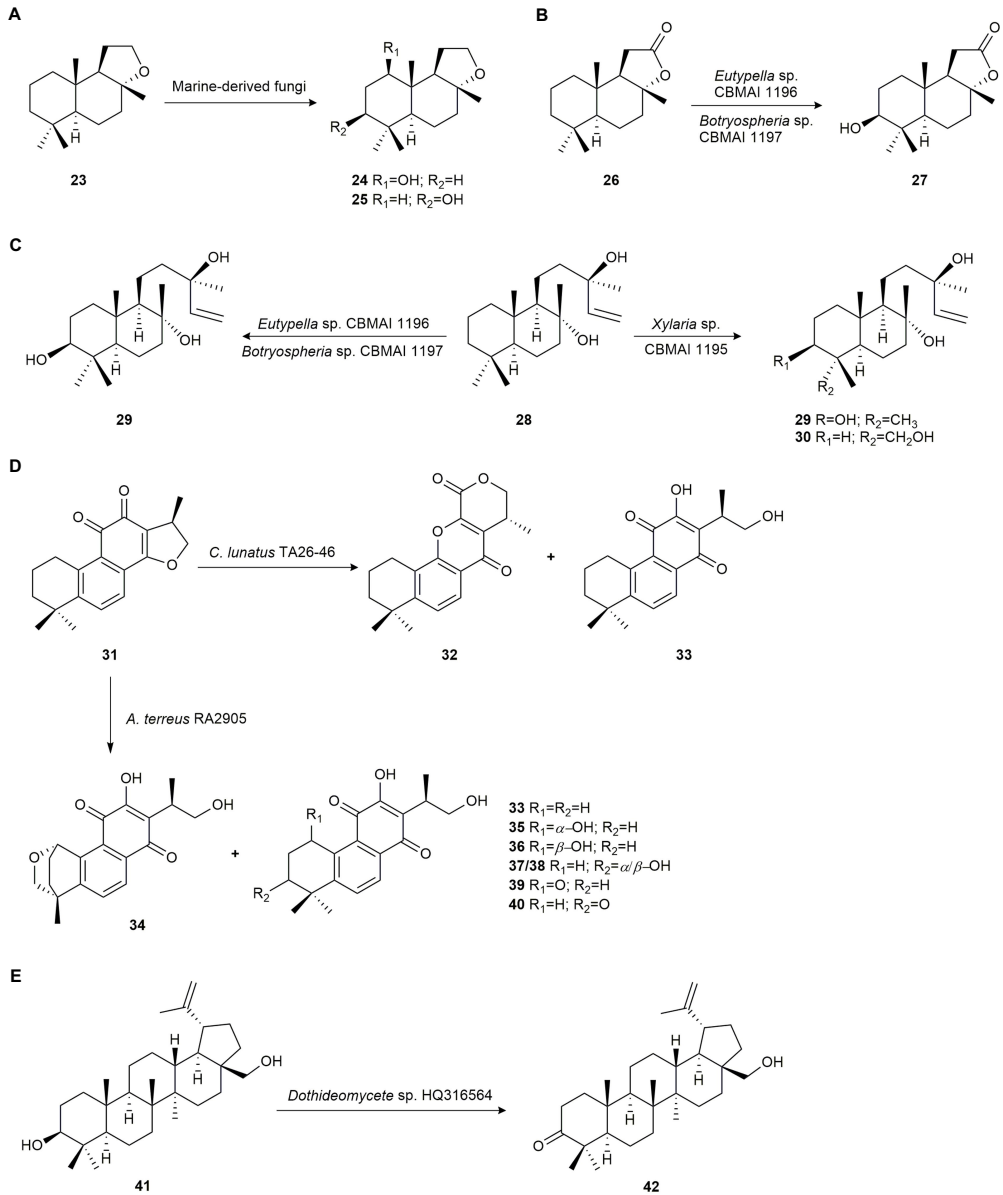
Terpene biotransformations. **(A)** Regioselective hydroxylation of (−)-ambrox (**23**) by *Aspergillus sydowii* CBMAI 934, *Eutypella* sp. CBMAI 1196 and *Botryosphaeria* sp. CBMAI 1197 (Martins et al., 2015). **(B)** Regioselective hydroxylation of (+)-sclareolide (**26**) by *Eutypella* sp. CBMAI 1196 and *Botryospharia* sp. CBMAI 1197 (Martins et al., 2015). **(C)** (−)-Sclareol (**28**) biotransformation by *Eutypella* sp. CBMAI 1196, *Botryospheria* sp. CBMAI 1197 and *Xylaria* sp. CBMAI 1195 (Martins et al., 2015). **(D)** Cryptotanshinone (**31**) biotransformation by *Cochliobolus lunatus* TA26-46 and *Aspergillus terreus* RA2905 (Wu et al., 2020). **(E)** Regioselectivity oxydation of betulin (**41**) by *Dothideomycete* sp. HQ31654 (Liu et al., 2013).

Some bromosesquiterpenes isolated from the Okinawan red alga *Laurencia luzonensis* were used as biotransformation substrates aiming at obtaining bioactive derivatives (Koshimura et al., 2009). Aplysistatin (**15**), with antileukenic activity, was converted into the new compounds **16–18** by *R. atrovirens* NRBC 32362, and into **19** by *Rhinocladiella* sp. K-001, marine-derived fungi isolated from the Okinawan brown alga *Stypopodium zonale.* The biotransformation reactions involved hydroxylation at C-5 or C-9, and the shift of the methyl group at C-11 to the C-10 position by *R. atrovirens* NRBC 32362, and stereoselective reduction of the C-C double bond at C-3 by *Rhinocladiella* sp. K-001 (Figure 1E). Moreover, palisadin A (**20**) was dehydrobrominated to 9,10-dehydrobromopalisadin A (**21**), and 12-hydroxypalisadin B (**22**) was converted into compounds **20** and **21** by *Rhinocladiella* sp. K-001 (Figure 1F; Koshimura et al., 2009).

(−)-Ambrox (**23**) is a natural product of animal origin with a high commercial value used in the fragrance industry. The biocatalytic potential of the marine-derived fungi *Aspergillus sydowii* CBMAI 934, *Eutypella* sp. CBMAI 1196 and *Botryosphaeria* sp. CBMAI 1197 was studied. The fungus *A. sydowii* CBMAI 934 was isolated from the marine sponge *Chelonaplysilla erecta,* while *Eutypella* sp. CBMAI 1196, and *Botryosphaeria* sp. CBMAI 1197 were isolated from the red marine alga *B. radicans*. These fungi were able to regioselectively hydroxylate compound **23** to 1*β*-hydroxy-ambrox (**24**) and (−)-3*β*-hydroxy-ambrox (**25**; Figure 2A; Martins et al., 2015).

The same authors used *Eutypella* sp. CBMAI 1196 and *Botryospharia* sp. CBMAI 1197 to biotransform (+)-sclareolide (**26**), a bioactive sesquiterpene with several pharmacological activities. Both microorganisms produced the hydroxylation of **26** in the 3*β*-position yielding 3*β*-hydroxy-sclareolide (**27**; Figure 2B). Yields were 7 and 34% for *Eutypella* sp. and *Botryospharia* sp., respectively (Martins et al., 2015).

(−)-Sclareol (**28**), a diterpene isolated from the essential oil of *Salvia sclarea*, exhibits antifungal and antibiotic activity and is used in the tobacco and fragrance industries. Biotransformation of this compound by the marine-derived fungus *Xylaria* sp. CBMAI 1195, isolated from red marine alga *B. radicans* collected in the South Atlantic Ocean, São Paulo, Brazil, yielded the hydroxylated products (−)-3*β*-hydroxy-sclareol (**29**) and (+)-18-hydroxy-sclareol (**30**) with 31 and 10% yield, respectively. However, *Eutypella* sp. CBMAI 1196 and *Botryosphaeria* sp. CBMAI 1197 only produced **29**, with 55 and 69% yield, respectively (Figure 2C; Martins et al., 2015).

The biotransformation of cryptotanshinone (**31**), a diterpene from the plant *Salvia miltiorrhiza* and effective in the treatment of cardiovascular and infectious diseases, was studied by Wu et al. (2020) to obtain novel bioactive derivatives using the marine-derived fungi *Cochliobolus lunatus* TA26-46 and *Aspergillus terreus* RA2905. These microorganisms were isolated from the zoanthid *Palythoa haddoni* and the sea hare *Aplysia pulmonica* collected from the Weizhou coral reef in the South China Sea, respectively. Biotransformation of **31** by *C. lunatus* TA26-46 yielded the rearranged product salviamone B (**32**), and the known compound neocryptotanshinone (**33**; Figure 2D).

Also, the new oxygenated compounds (1*S*,4*R*,15*R*)-1,18-epoxy-neocryptotanshinone (**34**), (1*S*,15*R*)-1-hydroxy-neocryptotanshinone (**35**), (1*R*,15*R*)-1-hydroxy-neocryptotanshinone (**36**), (15*R*)-3-hydroxy-neocryptotanshinone A (**37**), (15*R*)-3-hydroxy-neocryptotanshinone B (**38**), (15*R*)-1-keto-neocryptotanshinone (**39**), (15*R*)-3-keto-neocryptotanshinone (**40**), and metabolite **33** were obtained as biotransformation products of cryptotanshinone (**31**) from a culture media of *A. terreus* RA2905 (Figure 2D). Compounds **32**, **40** and **41** exhibited higher anti-bacterial activity against methicillin-resistant *Staphylococcus aureus* (MRSA) than **31** (Wu et al., 2020).

Betulin (**41**) and its derivatives are triterpenes with a wide range of biological activities. Liu et al. (2013) developed a biocatalytic method to obtain betulone (**42**) from betulin (**41**; Figure 2E) using the marine fungus *Dothideomycete* sp. HQ 316564, isolated from *Galaxea fascicularis* L. found in the South China Sea. This strain was able to regioselectively catalyze the oxidation of the hydroxyl group at C-3.

### 2.2. Steroids

Steroids are natural products with important physiological and biological activities. The great versatility of microorganisms in the production of highly valuable steroidal compounds for the pharmaceutical industry is well known (Bhatti and Khera, 2012).

**Figure 3:**
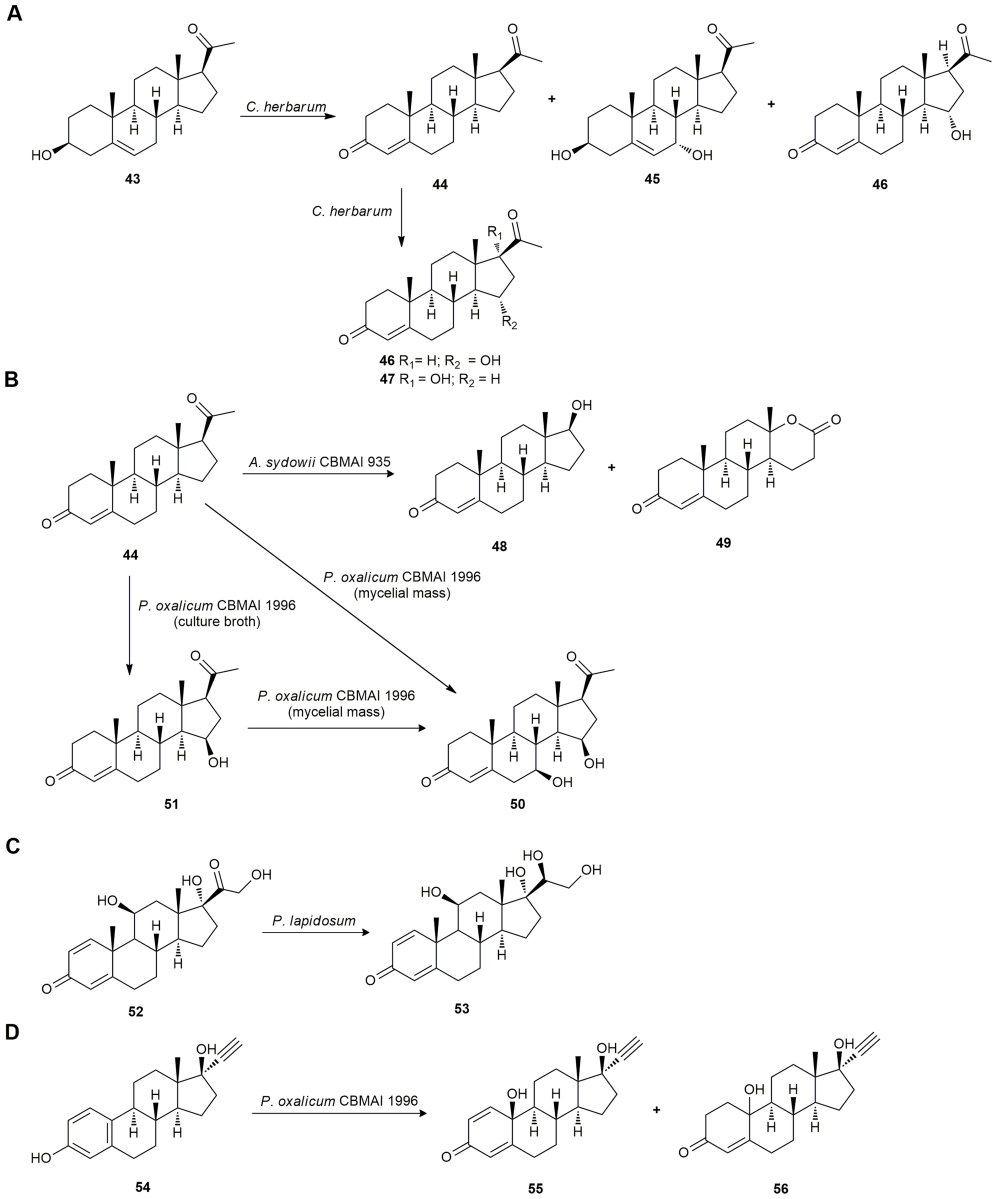
Steroid biotransformations. **(A)** Biotransformation of pregnenolone (**43**) and progesterone (**44**) by *Cladosporium herbarum* (Namboori et al., 1980). **(B)** Biotransformation of progesterone (**44**) by *Aspergillus sydowii* CBMAI 935 (de Paula and Porto, 2020) and by *Penicillium oxalicum* CBMAI 1996 (de Paula et al., 2021) **(C)** Prednisolone (**52**) reduction by *Penicillium lapidosum* SSW (Sultan et al., 2014). **(D)** Ehinylestradiol (**54**) bioconversion by *P. oxalicum* CBMAI 1996 (de Queiroz et al., 2020).

Transformation of pregnenolone (**43**) and progesterone (**44**) by the marine fungus *Cladosporium herbarum* using both mycelium and spores was studied. Using mycelium, the major products obtained with pregnenolone (**43**) as the substrate, were progesterone (**44**; 25%), 7*α*-hydroxypregnenolone (**45**; 10% yield), and 15*α*-hydroxyprogesterone (**46**; 20%); and using spores, the only major product was **45** (30%). When **44** was used as substrate, the major products obtained using mycelium and spores were **46** and 17*α* -hydroxyprogesterone (**47**; Figure 3A; Namboori et al., 1980).

Later, de Paula and Porto (2020) studied the biotransformation of progesterone (**44**) by *A. sydowii* CBMAI 935, isolated from the marine sponge *Geodia corticostylifera*. This fungus was able to oxidize progesterone (**44**) at the C17-site producing testosterone (**48**) and testololactone (**49**) as a mayor product in a good yield (Figure 3B). In addition, this bio-oxidation by means of a Baeyer-Villiger reaction showed that important enzymes are present in *A. sydowii* CBMAI 935 that assist in the biotransformation of related steroids.

The same authors carried out the progesterone (**44**) biotransformation using the marine-derived fungus *Penicillium oxalicum* CBMAI 1996, isolated from the marine sponge *C. erecta* collected in the South Atlantic Ocean, São Paulo (Brazil). The mycelia produced the steroid 7*β*,15*β*-dihydroxyprogesterone (**50**), and the culture broth of this fungus yielded the steroid 15*β*-hydroxyprogesterone (**51**) by hydroxylation at C-7 and C-15 *via* cytochrome P-450 monooxygenases. Also, compound **51** was biotransformed by the fungus mycelia to **50** (Figure 3B; de Paula et al., 2021).

While investigating the biocatalytic capacity of marine endophytic fungi, Sultan et al. (2014) studied the biotransformation of the anti-inflammatory drug prednisolone (**52**) as a model compound using *Penicillium lapidosum* SSW, isolated from an unidentified green alga from the West coast of Malaysia. The fermentation of **52** yielded only 20*β*-hydroxyprednisolone (**53**), proving its stereoselective reducing capacity (Figure 3C).

Twelve marine-derived fungal strains were tested for their potential to perform bio-oxidation reactions using ethinylestradiol (**54**) as substrate. Lastly, *P. oxalicum* CBMAI 1996 was selected, producing products **55** and **56** in poor yield (10 and 6%, respectively; Figure 3D). Biotransformation pathway of **54** suggests the presence of several enzymes such as phenol oxidases, monooxygenases, and ene-reductases in the strain CBMAI 1996. Also, kinetic monitoring of the biotransformation of ethinylestradiol (**54**) was performed and revealed that the yields of **55** and **56** could not be maximized over time, although the conversion was approximately 100%. This indicates that a biodegradation process was occurring at the same time as the biotransformation. It is worth noting that marine-derived fungi can play a relevant role in the biodegradation of steroidal compounds in the ocean and in wastewater due to their inappropriate disposal (de Queiroz et al., 2020).

### 2.3. Polyketides

Polyketides are a structurally and functionally diverse family of natural products with a wide range of bioactivities and have become a rich source of new pharmaceutical compounds (Ridley and Khosla, 2009). Hence, compounds like these may be good candidates for modification by biotransformation to enhance their biological activity.

**Figure 4:**
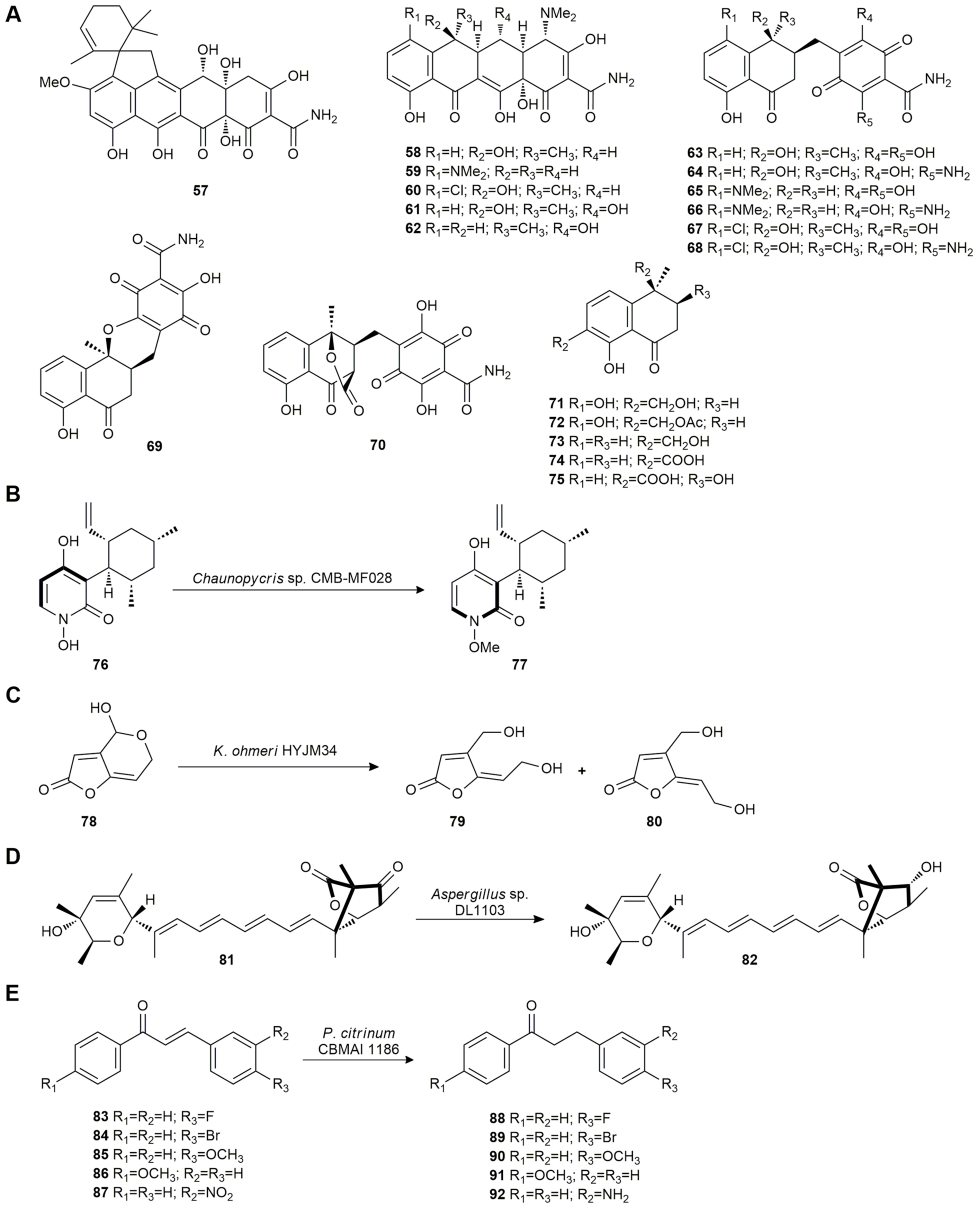
Polyketide biotransformations. **(A)** Production of *seco*-cyclines (**63–70**) from tetracycline (**58**), minocycline (**59**), chlortetracycline (**60**), and *hemi*-cyclines (**71–75**) from oxytetracycline (**61**) and doxycycline (**62**) by *Paecilomyces* sp. CMB-MF010 (Shang et al., 2016). **(B)** Biotransformation of pyridoxatin (**76**) 77 by *Chaunopycris* sp. CMB-MF028 (Shang et al., 2017). **(C)** Patulin (**78**) bioconversion by *Kodameae ohmeri* HY JM34 (Dong et al., 2015). **(D)** Reduction of wortmannilactone *F* (**81**) by *Aspergillus* sp. DL1103 (Liu et al., 2018d). **(E)** Reduction of chalcones **83–87** by *Penicillium citrinum* CBMAI 1186 (Ferreira et al., 2014).

Viridicatumtoxin A (**57**) is a tetracycline-like antibiotic isolated from *Paecilomyces* sp. CMB-MF010, an Australian marine mollusk-associated fungus. Considering that 5-oxotetracyclines may exhibit enhanced anti-vancomycin-resistant *Enterococci* (VRE) properties, Shang et al. (2016) studied the activity of viridicatumtoxin pharmacophore by biotransforming the commercial antibiotics tetracycline (**58**), minocycline (**59**), chlortetracycline (**60**), oxytetracycline (**61**) and doxycycline (**62**) using the fungus *Paecilomyces* sp. CMB-MF010. Fermentation of these antibiotic compounds yielded eight *seco*-cyclines (**63–70**) from **58**, **59** and **60**, and five *hemi*-cyclines (**71**–**75**) from **61** and **62** (Figure 4A). None of the biotransformation products exhibited antibiotic properties. These two mechanisms suggest a basis for predicting fungal biotransformation products for other members of the tetracycline family of antibiotics.

Later, Shang et al. (2017) also studied the biotransformative potential of *Chaunopycris* sp. CMB-MF028 and *Trichoderma hamatum* CMB-MF030, fungal strains co-isolated from the mollusk *Siphonaria* sp. In this report, co-cultivation of these strains was used to activate silent biosynthetic gene clusters leading to the biotransformation and deactivation, using *Chaunopycris* sp. CMB-MF028, of the antifungal pyridoxatin (**76**) produced by *T. hamatum* CMB-MF030, to methyl-pyridoxatin (**77**; Figure 4B), without activity.

Patulin (**78**) is a toxic compound produced by a wide range of fungi. This secondary metabolite is toxic to humans and animals at very low doses and can be found in fruits, vegetables, cereals and shellfish. As physical and chemical methods have proven to be limited (high cost, safety issues…), biological control is proposed as an alternative for patulin detoxification. In 2015, Dong et al. (2015) screened 58 marine yeast strains for their capacity to biodegrade patulin (**78**). The *Kodameae ohmeri* HYJM34 strain proved to be the most efficient yielding (*E*)-ascladiol (**79**) and (*Z*)-ascladiol (**80**) as degradation products (Figure 4C), which are less toxic than patulin (**78**). The yeast strain was also tested in apple juice supplemented with a patulin (**78**) microenvironment (attempting to imitate real conditions), showing a high degree of contaminant degradation.

Due to the emergence of parasites resistant to anti-helminthics, new parasite-specific pest control products that are minimally toxic to the host are urgently needed. In 2018, Liu et al. (2018d) studied the biotransformation of wortmannilactone *F* (**81**), a selective inhibitor of NADH-fumarate reductase and NADH-rhodoquinone reductase, using the marine-derived *Aspergillus* sp. DL1103 to improve its anti-helminthic activity. This biotransformation yielded wortmannilactone M (**82**) through a reduction at the C-3 carbonyl group (Figure 4D). Tests of its anti-helminthic activity showed that it significantly reduced the number and size of *Trichinella spiralis* in the intestinal tissue of mice *in vivo.*

Chalcones are precursors or components of many natural compounds such as flavonoids and isoflavonoids and have several medical applications such as anti-cancer, anti-ulcer, anti-tuberculosis, anti-inflammatory, and anti-bacterial treatments (Thapa et al., 2021).

Reduction of the double bond of chalcone produces dihydrochalcones, which exhibit several biological activities such as cytotoxic, antileishmanial, antitumor, antibacterial, anti-*Trypanosoma cruzi* and anti-HIV. Considering that direct hydrogenation of chalcones by chemical methods is relatively difficult, Ferreira et al. (2014) studied the reduction of several analogous of chalcones using *Penicillium citrinum* CBMAI 1186, isolated from the marine alga *Caulerpa* sp. collected off the coast of the State of São Paulo, Brazil. Compounds **83**–**87** were reduced to the corresponding dihydrochalcones **88**–**92** in good yields (Figure 4E).

**Figure 5:**
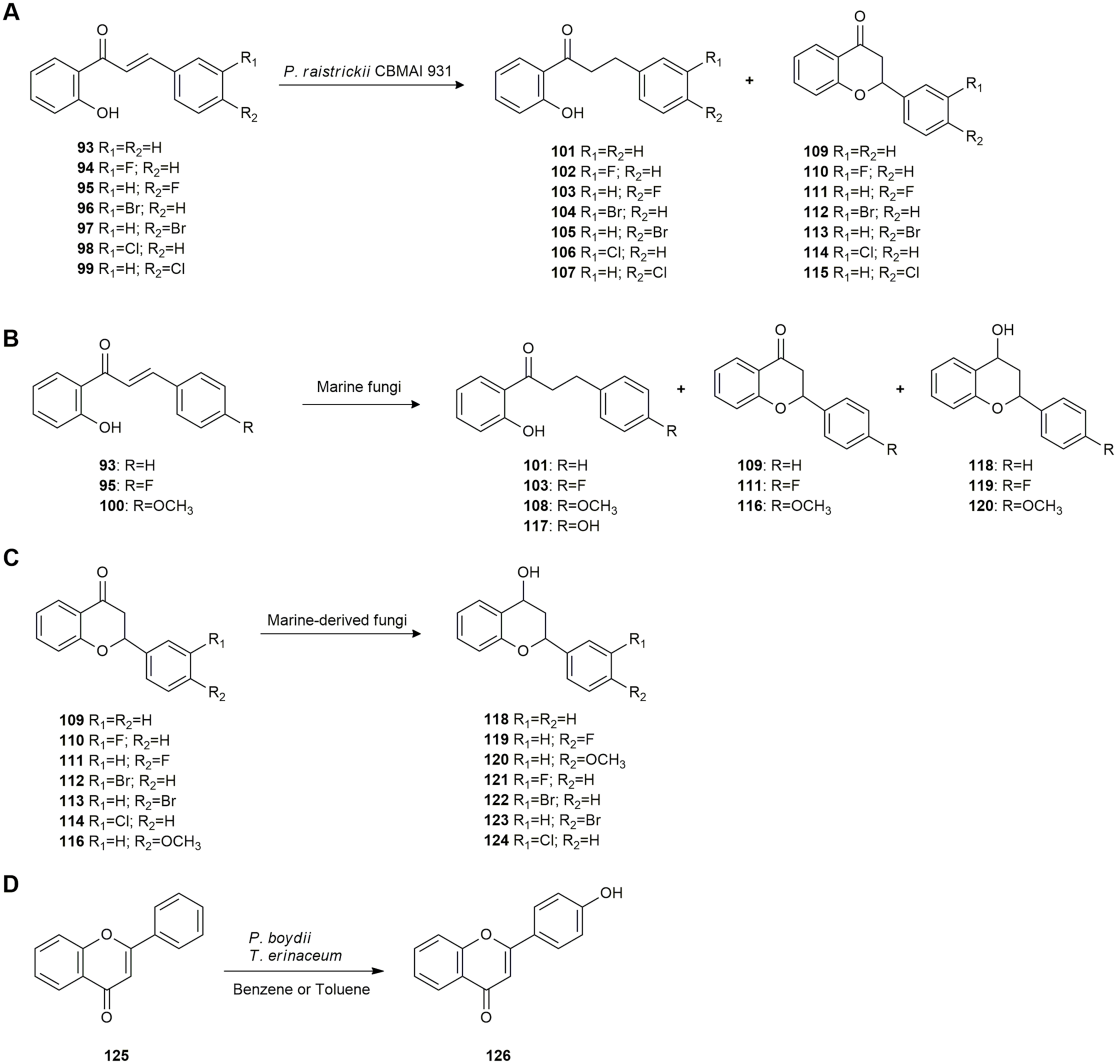
Polyketide biotransformations. **(A)** Hydrogenation of halogenated 2′-hydroxychalcones **93–99** by *Penicillium raistrickii* CBMAI 935 (de Matos et al., 2019). **(B)** Biotransformation of 2′-hydroxychalcones **93**, **95** and **100** by marine fungi (de Matos et al., 2021b). **(C)** Flavone reduction by marine fungi (de Matos et al., 2021a). **(D)** Regioselective biotransformation of flavone **125** by *Pseudallescheria boydii* and *Trichoderma erinaceum* (Kunteng et al., 2015).

Subsequently, the chemoselective hydrogenation of halogenated 2′-hydroxychalcones (**93–99**) was studied by de Matos et al. (2019). The marine-derived fungus *Penicillium raistrickii* CBMAI 931 was chosen as the biocatalyst and biotransformation conditions were optimized. Halogenated 2′-hydroxy-dihydrochalcones **101–107** were obtained at a good conversion rate (78–99%) with moderate isolated yields (31–65%), indicating that this fungus possesses ene-reductase enzymes that efficiently transform chalcones with chemo- and regioselective control. Bromo- and fluoro-substituted chalcones exhibited higher conversion rates than chloro-substituted compounds. In all cases, the formation of flavones **109–116** was low (Figure 5A).

In 2021, the same authors used several marine-derived fungi to biotransform the 2′-hydroxychalcones **93**, **95** and **100**. The main reactions involved hydrogenation of the double bond, hydroxylation of the B-ring, and cyclization to obtain flavanones. *P. raistrickii* CBMAI 931 catalyzed a chemoselective reduction to produce the corresponding 2′-hydroxydihydrochalcones **101**, **103** and **108**. *A. sydowii* CBMAI 935 promoted hydroxylation to yield 2′,4-dihydroxy-dihydrochalcone (**117**) only from **93** at a yield of 26%. The reaction caused by *P. citrinum* CBMAI 1186 and *Mucor racemosus* CBMAI 847 yielded cyclization products without enantioselectivity (**118–120**; Figure 5B; de Matos et al., 2021b).

Flavanones are a type of flavonoids featuring interesting biological activities and are found at high concentrations in many fruits such as grapes, oranges, lemons, and some aromatic herbs. Thus, given the potential of marine fungi for the asymmetric reduction of natural products, de Matos et al. (2021a) used different marine-derived fungi to reduce halogenated flavanones **109**–**114** and **116** to flavan-4-ols **118**–**124** with high enantiomeric excesses of both *cis*- and *trans*-diastereoisomers (Figure 5C). The strains *Cladosporium* sp. CBMAI 1237 and *Acremonium* sp. CBMAI 1676 were used and high yields were obtained. This was the first report of brominated flavon-4-ols.

In this work, flavanone **109** was biotransformed by the strain *P. raistrickii* CBMAI 931 resulting in the production of dihydrochalcone **101** as the only product. Compound **101** and the dihydroxy dihydrochalcone **117** were produced by *A. sydowii* CBMAI 935 and *Fusarium* sp. CBMAI 1830 with **109** as substrate. This is interesting because in most cases the opening of the C ring in flavanones leads to the formation of chalcones, but this formation was not observed in this case (de Matos et al., 2021a).

Kunteng et al. (2015) studied the biotransformation of flavone (**125**) by the marine fungi *Pseudallescheria boydii* and *Trichoderma erinaceum*, which exhibited high tolerance to the aromatic hydrocarbons benzene and toluene. 4’-Hydroxy flavone (**126**) was the only product and was obtained regioselectively at high yield (Figure 5D). In this work, the hydroxylases activated by the biodegradation of benzene and toluene appear to be responsible for the biotransformation.

### 2.4. Other natural products

One of the most important scaffolds for drug discovery is the indole core, present in many approved drugs due to their ability to mimic the structure of peptides. This allows them to bind reversibly to enzymes which opens the door to the discovery of novel drugs with different modes of action (Zhang et al., 2015).

**Figure 6:**
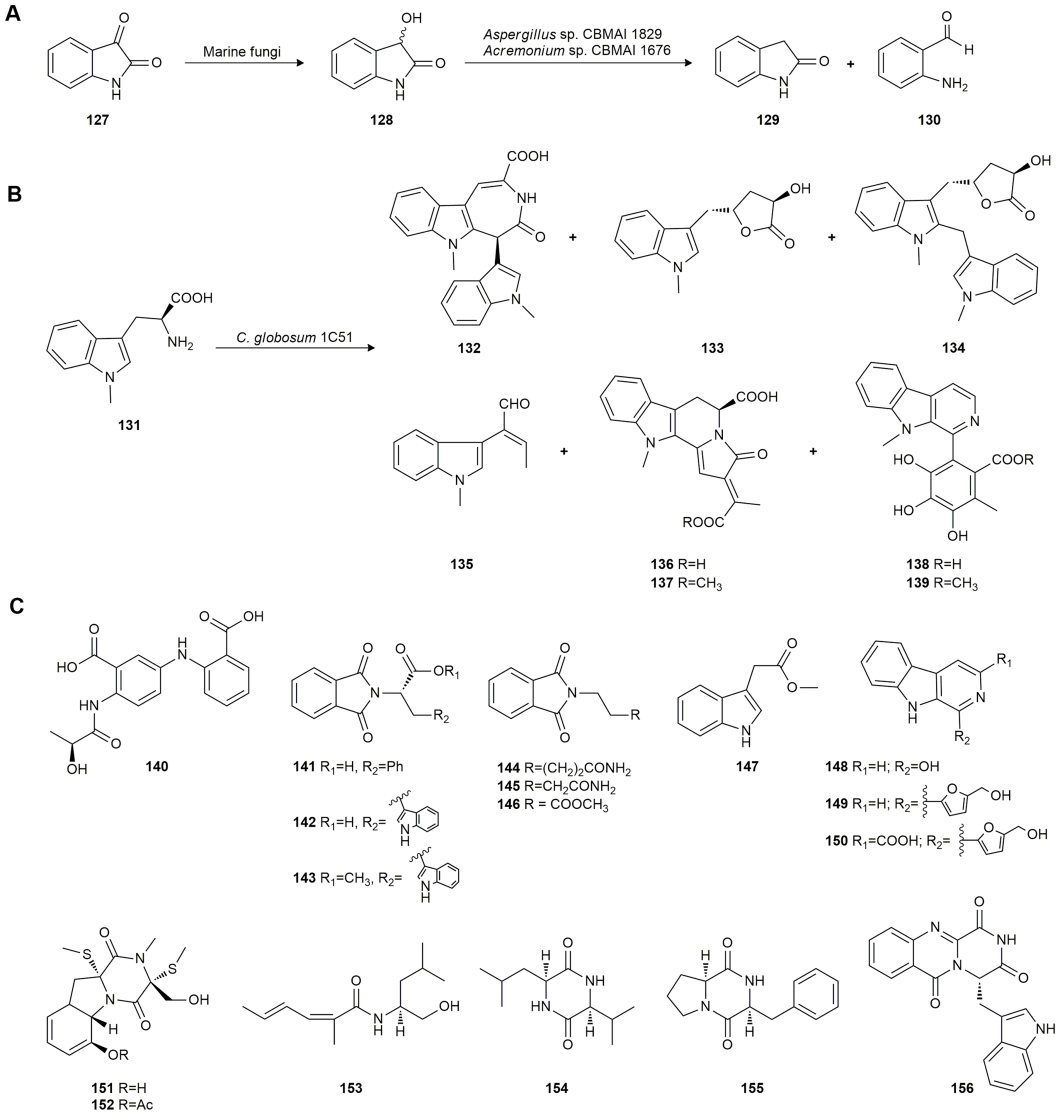
Biotransformations of other natural products. **(A)** Isatin (**127**) biotransformation by marine fungi (Birolli et al., 2017). **(B)** 1-Methyl-l-tryptophan (**141**) biotransformation by *Chaetomium. globosum* 1C51 (Yan et al., 2019). **(C)** Biotransformation products of a mixture of anthranilic acid and phthalimide by *Scedosporium apiospermum* F41-1 (Chen et al., 2021).

Birolli et al. (2017) studied the bioreduction of isatin (**127**), a metabolic derivative of adrenaline whose derivatives exhibit diverse pharmacological properties, to produce dioxindole (**128**). This compound has shown pharmacological potential, i.e., antiallergic, anti-inflammatory and anticancer activity. These authors tested marine-derived fungi isolated from marine sponges collected at São Sebastião off the coast of São Paulo (Brazil) and found that they yielded the enantioenriched dioxindole (**128**) by asymmetric bioreduction (Figure 6A). All strains managed to biotransform **127** but *Penicillium miczynskii* CBMAI 930, *Trichoderma* sp. CBMAI 932, *Aspergillus* sp. CBMAI 1829 and *Acremonium* sp. CBMAI 1676 showed a good conversion rate (63–83%) and modest selectivity to *S* enantiomer (42–48% *ee*). In contrast, *M. racemosus* CBMAI 847, *A. sydowii* CBMAI 935 and *Fusarium* sp. CBMAI 1830 did not efficiently convert the xenobiotic substrate.

Further experiments with *Aspergillus* sp. CBMAI 1829 and *Acremonium* sp. CBMAI 1676 revealed the production of small amounts of indolin-2-one (**129**) and 2-aminobenzaldehyde (**130**) as biodegradation products from **127** and **128** (Figure 6A), probably used as carbon and nitrogen sources due to the absence of nutrients. Previous studies indicate that one of the most common metabolic pathways in the biotransformation of **127** is its hydrolysis by isatin hydrolase, a manganese-dependent enzyme (Bjerregaard-Andersen et al., 2014). Both experiments produced a decrease in the conversion of isatin (**127**) indicating that the biodegradation and biotransformation processes occur simultaneously.

Later, Yan et al. (2019) studied the biotransformation of 1-methyl-l-tryptophan (**131**) using the marine fish-derived fungus *Chaetomium globosum* 1C51 collected from the Yellow Sea, Yancheng City, China. Fermentation, initially intended to activate silent genes, yielded six novel (**132–137**) and two known (**138–139**) chaetogline indole alkaloids (Figure 6B). All isolated indols were evaluated for antimicrobial activity against pathogenic fungi and bacteria with agricultural interest and it was found that compounds **132**, **134**, **136** and **138** were very active against the rice-pathogenic bacteria *Xanthomonas oryzae* pv. *oryzae* and *X. oryzae* pv. *oryzicola*, and **138** inhibited the pathogenic fungus *Sclerotinia sclerotiorum,* responsible for rape sclerotinia rot.

To obtain new indol alkaloids with interesting pharmacological activity, a mixture of anthranilic acid and phthalimide were used as substrates in biotransformation by the marine fungus *Scedosporium apiospermum* F41-1, isolated from the soft coral *Lobophytum crissum* in China. A new diphenylamine derivative (**140**) and 16 known alkaloids (**141**–**156**) were isolated and identified (Figure 6C). Evaluation of their lipid-lowering property revealed their potential as drug candidates to treat hyperlipidemia, mainly compound **149**. None of them were cytotoxic (Chen et al., 2021).

**Figure 7:**
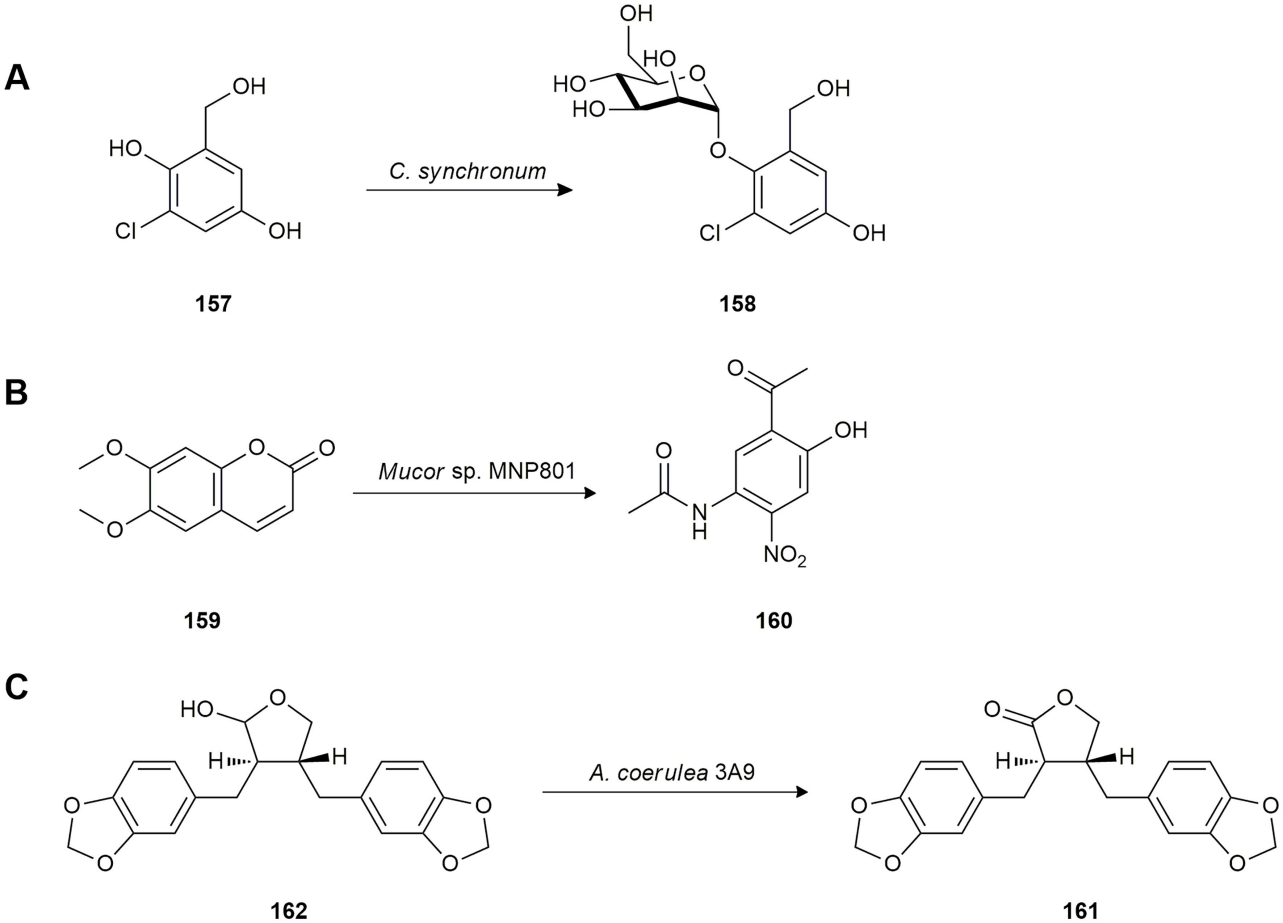
Biotransformations of other natural products. **(A)** Chlorogentisyl alcohol (**157**) mannosidation by *Chrysosporium synchronium* (Yun et al., 2011). **(B)** Scoparone (**159**) biotransformation by *Mucor* sp. MNP801 (Wang et al., 2010). **(C)** Hinokinin (**161**) production from cubetin (**162**) by *Absidia coerulea* 3A9 (de Souza et al., 2021).

Chlorogentisyl alcohol (**157**) is an antitumoral natural product isolated from the marine algicolous fungus *Aspergillus* sp. While screening marine microorganisms able to biotransform bioactive metabolites, the bioconversion of **157** was studied using the marine-derived fungus *Chrysosporium synchronium* isolated from the surface of edible brown alga *Sargassum ringgoldium* collected in Korea. Fermentation yielded the new glycoside 1-*O*-(*α*-d-mannopyranosyl)chlorogentisyl alcohol (**158**) as a result of mannosidation (Figure 7A). The antioxidant activity of both compounds (**157** and **158**) was evaluated and significant radical-scavenging activity against 1,1-diphenyl-2-picrylhydrazyl radical (DPPH) was revealed, even higher than the positive control (Yun et al., 2011).

Scoparone (**159**) is a simple coumarin from the botanical drugs of *Artemisia* species used in traditional Chinese medicine. To explore new active derivatives, Wang et al. (2010) studied the biotransformation of this coumarin using the marine fungus *Mucor* sp. MNP801 which yielded 2-acetyl-4-acetylamino-5-nitrophenol (**160**; Figure 7B).

Given the therapeutic potential as a trypanosomicidal, anti-inflammatory and analgesic agent of hinokinin (**161**), the oxidized form of cubetin (**162**), de Souza et al. (2021) investigated the biotransformation of **162** by the marine fungus *Absidia coerulea* 3A9, isolated from the ascidian *Distaplia stilyfera*, to improve the production of hinokinin (**161**; Figure 7C).

## 3. Biotransformation of cyano compounds

Organonitriles are considered an important class of compounds for the chemical industry because of their use as intermediates in the synthesis of carboxylic acids, esters, amines, amides, amidines, aldehydes, ketones, and heterocyclic compounds. Their chemical transformation usually involves pollutant compounds, so the study of the biocatalytic potential of marine fungi enzymatic systems is an interesting environmentally-friendly way to apply them in bioremediation.

de Oliveira et al. (2013) tested the potential of 12 marine-derived fungi for the biotransformation of phenylacetonitrile. The strains used were: *A. sydowii* Gc12 and *P. miczynskii* Gc5, collected from marine sponge *G. corticostylifera*, *A. sydowii* Ce19, *A. sydowii* Ce15, *Bionectria* sp. Ce5 and *P. raistrickii* Ce16, from marine sponge *C. erecta*, *A. sydowii* Dr.(M3)2, *P. raistrickii* Dr.(B)2, *Penicillium decaturense* Dr.(F)2 and *Cladosporium* sp. Dr.(A)2, from marine sponge *Dragmacidon reticulatum*, and *P. oxalicum* F30 and *P. citrinum* F53, from marine algae *Caulerpa* sp. Sponges and algae were collected in the South Atlantic Ocean off the northern coast of the state of São Paulo, Brazil. The presence of phenylacetonitrile was tolerated by eight strains and its bioconversion produced 2-hydroxyphenylacetic acid at a yield of 51%, an aromatic compound used as an intermediate in the preparation of biologically active products in the pharmaceutical industry such as antihypertensive agents. The bioconversion of 4-fluorophenylacetonitrile to 4-fluorophenylacetic acid was also tested to study the enzymatic pathway employing *A. sydowii* Ce19.

The same authors conducted further experiments to study the bioconversion of several methylphenylacetonitriles by the marine filamentous fungus *A. sydowii* CBMAI 934, isolated from the marine sponge *C. erecta*, to afford the corresponding acids in a very good yield. In this experiment, phenylacetonitrile was employed as a carbon and nitrogen source, inducing the arylaliphatic nitrilases. The marine fungus *A. sydowii* CBMAI 934 was thus shown to be a potential biocatalyst for the synthesis of carboxylic acids from nitriles (de Oliveira et al., 2014).

**Figure 8:**
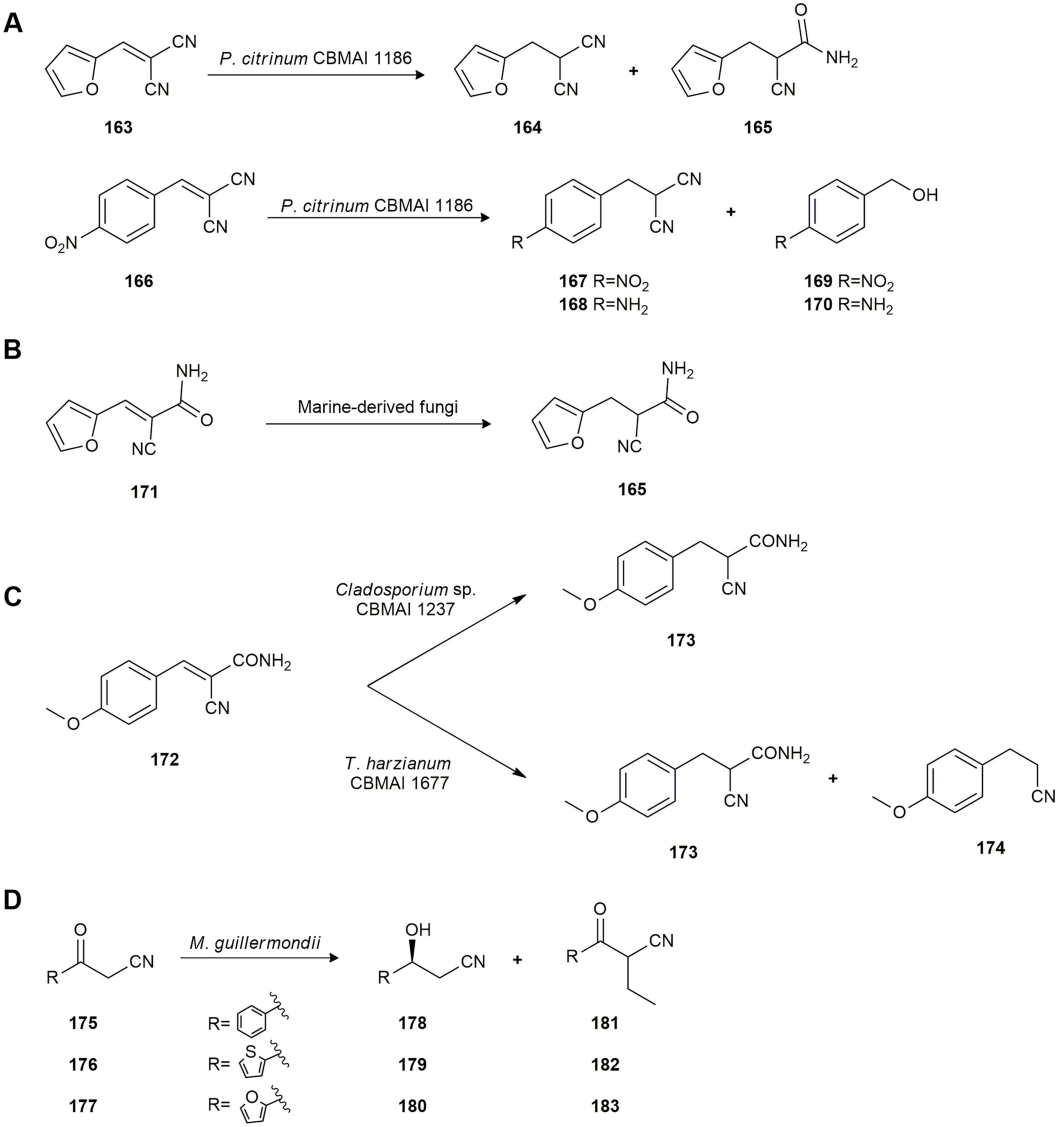
Biotransformations of cyano compounds. **(A)** Bioconversion of compounds **163** and **166** by *Penicillium citrinum* CBMAI 1186 (Jimenez et al., 2016). **(B)** Hydrogenation of *E*-2-cyano-3-(furan-2-yl)acrylamide (**171**) by *P. citrinum* CBMAI 1186, *Trichoderma* sp. CBMAI 932 and *Aspergillus sydowii* CBMAI 935 (Jimenez et al., 2019). **(C)** Reduction of (*E*)-2-cyano-3-(4-methoxyphenyl)acrylamide (**172**) by marine-derived fungi (Birolli et al., 2020). **(D)** Reduction of 3-oxo-3-arylpropanenitriles **175–177** by *Meyrozyma guillermondii* UBOCC-A-214008 (Serra et al., 2016).

Chemoselective reduction of the C-C double bond of aromatic malonitrile derivatives can have applications as intermediates in the organic synthesis of pharmaceutical molecules. In this connection, Jimenez et al. (2016) studied the biotransformation of aromatic malonitriles prepared by Knoevenagel condensation using whole cells of the marine-derived fungus *P. citrinum* CBMAI 1186. The fungus catalyzed the chemoselective bioreduction of every Knoevenagel adduct generally with good yield. In this case, success depended on the electronic effect promoted by the substituent linked to the aromatic ring. Therefore, the bioconversion of compound **163** into **164** and **165** exhibited ene-reductase and nitrile hydratase activity induced by the substrate. Compound **166**, which has an electron withdrawing substituent (-NO_2_), followed a completely different catalytic pathway as its biotransformation yielded a mixture of products (**167–170**; Figure 8A).

Jimenez et al. (2019) also studied the enantioselective ene-reduction of *E*-2-cyano-3-(furan-2-yl)acrylamide (**171**) by the marine-derived fungi *P. citrinum* CBMAI 1186 and *Trichoderma* sp. CBMAI 932, isolated from the sponge *G. corticostylifera*, and *A. sydowii* CBMAI 935, from the marine sponge *C. erecta*. Sponges were collected from Sao Sebastiao City, on the coast of the State of São Paulo, Brazil. Biotransformation by *P. citrinum* CBMAI 1186 yielded (*S*)-2-cyano-3-(furan-2-yl)propanamide (*S*-**165**) with 99% *ee*, and (*R*)-2-cyano-3-(furan-2-yl)propanamide (*R*-**165**) was produced by *A. sydowii* CBMAI 935 and *Trichoderma* sp. CBMAI 932 with 97 and 98% *ee*, respectively (Figure 8B), with high yields.

Birolli et al. (2020) also studied the biocatalytic ene-reduction of Knoevenagel adducts employing the following eight marine-derived fungi strains: *A. sydowii* CBMAI 935, *Cladosporium* sp. CBMAI 1237, *Microsphaeropsis* sp. CBMAI 1675, *Acremonium* sp. CBMAI 1676, *Westerdykella* sp. CBMAI 1679, *P. citrinum* CBMAI 1186, *P. oxalicum* CBMAI 1185 and *Trichoderma harzianum* CBMAI 1677, isolated from several sponges and algae collected from the South Atlantic Ocean at São Paulo, Brazil. A preliminary biotransformation experiment by *Cladosporium* sp. CBMAI 1237 using the adduct (*E*)-2-cyano-3-(4-methoxyphenyl)acrylamide (**172**) as substrate yielded 2-cyano-3-(4-methoxyphenyl)propanamide (**173**) showing that strain CBMAI 1237 had an excellent yield of 95% with 96% conversion in 16 h. Further experiments with the strain *T. harzianum* CBMAI 1677 and substrate **172** showed that better results could be obtained employing a different phosphate buffer with hexane, yielding compounds **173** and **174** (Figure 8C). However, *Cladosporium* sp. CBMAI 1237 is a greener methodology since hexane is not used. Hence, several more Knoevenagel adducts were tested employing *Cladosporium* sp. CBMAI 1237 with a view to shorter reaction times, showing good yields but no enantioenriched compounds were obtained due to a ketenimine equilibrium which racemized the products.

Chiral *β*-hydroxy nitriles are valuable intermediates for the synthesis of several biologically active compounds. Thus, Serra et al. (2016) evaluated the reduction of several 3-oxo-3-arylpropanenitriles (**175**–**177**) with several marine yeasts isolated from deep sub-seafloor sediments in New Zealand. Strains *Rhodotorula mucilaginosa* UBOCC-A-214025 and *R. mucilaginosa* UBOCC-A-214036 yielded bioreduction products as the only compounds of biotransformation affording the enantiomerically pure (*S*)-alcohols **178–180**, with very high enantioselectivity. However, *M. guillermondii* UBOCC-A-214008 afforded the aromatic ketones **181–183** as major products and only traces of the (*S*)-alcohols **178–180**, suggesting the presence of pyruvate decarboxylase activity in this strain (Figure 8D).

Continuing with the biotransformation of nitriles, Serra et al. (2019) tested the marine-derived yeast strain *M. guillermondii* LM2 (UBOCC-A-214008) for its ability to hydrolyse different aromatic, aliphatic and arylaliphatic nitriles employing cyclohexanecarbonitrile as the sole nitrogen source and nitrilase inductor. A two-step one-pot method for obtaining cells of LM2 strain endowed with high nitrilase activity was established. The yeast nitrilase system of LM2 strain was able to hydrolyse several nitrile substrates yielding the corresponding acids with good activity and high yields but exhibiting poor amidase activity. Shorter bioconversion times were achieved with the nitrilase induction strategy.

## 4. Biotransformation of carbonyl compounds

Biocatalytic asymmetric reduction of ketones is an important methodology for the preparation of enantiomerically enriched secondary alcohols which are valuable intermediates in the synthesis of pharmaceutical compounds of interest.

Chiral chlorohydrins are used as intermediates in the synthesis of various compounds exhibiting biological activity. Thus, Rocha et al. (2009) used various types of acetophenones as biotransformation substrates. They first studied the biotransformation of *α*-chloroacetophenone by several marine fungi and the asymmetric reduction of 2-chloro-1-phenylethanone into (*S*)-(−)-2-chloro-1-phenylethanol with different yields and enantiomeric excesses. Only fungi cultured in artificial sea water-based medium with a high concentration of chloride ions were able to reduce the substrate. Marine fungi were isolated from the sponges *G. corticostylifera* and *C. erecta*, collected off the coast at São Sebastião in the north of the state of São Paulo, Brazil.

Morais et al. (2018) reported the chemical synthesis of *α*-chloroketones, *in situ*, followed by reduction with whole cells of marine-derived fungi. *P. citrinum* CBMAI 1186, *P. oxalicum* CBMAI 1185, *A. sydowii* CBMAI 935*, P. raistrickii* CBMAI 931 and *M. racemosus* CBMAI 847 were used for the reduction of *α*-chloroacetophenones rendering (*S*)-alcohols. The conversions and enantiomeric excesses varied according to the substrate structures and the fungus used.

Rocha et al. (2010) investigated the biotransformation of *α*-bromoacetophenones by the marine fungus *A. sydowii* Ce19. This fungus catalyzed the bioconversion of *α*-bromoacetophenone into (*R*)-2-bromo-1-phenylethanol (56%), together with *α*-chlorohydrin (9%), 1-phenylethan-1,2-diol (26%), phenylethanol (5%) and acetophenone (4%). Moreover, the substituted *p*-bromo-*α*-bromoacetophenone and *p*-nitro-*α*-bromoacetophenone were biotransformed by the fungus affording a complex mixture of degradation products at low concentration.

Enantiomerically pure *β*-azido alcohols are precursors of chiral aziridines and amino alcohols used in the organic synthesis of bioactive molecules. Thus, Rocha et al. (2015) investigated the stereoselective biotransformation of four *α*-azido ketones to the corresponding *β*-azidophenylethanols using whole cells of seven strains of marine-derived fungi: *A. sclerotiorum* CBMAI 849, *C. cladosporioides* CBMAI 857, *P. raistrickii* CBMAI 931, *P. citrinum* CBMA 1186, *M. racemosus* CBMAI 847, *Beauveria felina* CBMAI 738, and *P. oxalicum* CBMAI 1185. In general, these fungi exhibited good potential as biocatalysts for this type of compound, yielding Prelog or anti-Prelog alcohols with good conversions and selectivities. The best results were achieved by *A. sclerotiorum* CBMAI 849, *C. cladosporioides* CBMAI 857, *P. raistrickii* CBMAI 931, and CBMA 1186, which catalyzed the bioreduction of 2-azido-1-(4-methoxyphenyl)ethanone to the corresponding (*R*)-2-azido-1-(4-methoxyphenyl)ethanol with excellent enantiomeric excesses (>99% *ee*). This work was the first screening of marine-derived fungi for the asymmetric reduction of *α*-azido ketones.

Several years later, Alvarenga and Porto (2017) tested the biocatalytic potential of five marine-derived fungi strains (*A. sydowii* CBMAI 935, *M. racemosus* CBMAI 847, *P. miczynskii* CBMAI 930, *Botryosphaeria* sp. CBMAI 1197 and *Hydropisphaera* sp. CBMAI 1194) to reduce 2-azido-1-phenylethanone and some derivatives to the corresponding alcohols to apply in the synthesis of enantiomerically enriched bioactive *β*-hydroxy-1,2,3-triazoles. A comparative study showed that all the strains were able to catalyze the bioreduction of 2-azido-1-phenylethanone to 2-azido-1-phenylethanol, but only *A. sydowii* CBMAI 935 and *M. racemosus* CBMAI 847 exhibited very high conversion and stereoselectivity values. Thus, both strains were used to biotransform 2-azido-1-(4-methoxyphenyl)ethanone, 2-azido-1-(4-bromophenyl)ethanone and 2-azido-1-(4-chlorophenyl)ethanone by asymmetric reduction into the corresponding alcohols with good yield. *A. sydowii* CBMAI 935 yielded (*S*)-2-azido-1-phenylethanols with high selectivity, and *M. racemosus* CBMAI 847 led to (*R*)-enantiomer for 2-azido-1-phenylethanone and methoxy- and bromo-derivatives, and (*S*)-enantiomer for the chloro-derivative.

The asymmetric bioconversion of *o*-, *m*- and *p*-iodoacetophenone was done with the marine-derived fungi *A. sclerotiorum* CBMAI 849, *A. sydowii* Ce19, *B. felina* CBMAI 738, *M. racemosus* CBMAI 847, *P. citrinum* CBMAI 1186, *P. miczynskii* Ce16, *P. miczynskii* Gc5, *P. oxalicum* CBMAI 1185, and *Trichoderma* sp. Gc1. All fungi were isolated from the sponges *G. corticostylifera* and *C. erecta*, the marine algae *Caulerpa* sp. and Brazilian cnidarian species, namely *Palythoa variabilis*, *Palythoa caribaeorum*, and *Mussismilia hispida*. *o*-Iodoacetophenone and *m*-iodoacetophenone were reduced to the corresponding (*S*)-alcohols, while *p*-iodoacetophenone was reduced to (*S*)- or (*R*)-*p*-iodophenylethanol depending on the fungus employed, mostly with excellent optical purity (Rocha et al., 2012c).

The same substrates were biotransformed by Mouad et al. (2012) using marine fungi isolated from the algae *B. radicans* and *Sargassum* sp. *Botryosphaeria* sp. Br-09 catalyzed the reduction of *o*- and *p*-iodoacetophenones with high optical purity (>99% *ee*) and excellent conversions but in the case of *Xylaria* sp. Br-61, only small yields of iodophenylethanols were rendered.

Competitive oxidation–reduction reactions were also observed in the bioconversion of *p*-iodophenylethanol by the marine fungus *P. oxalicum* CBMAI 1185. 4-Hydroxy-3-iodoacetophenone was biotransformed by *P. oxalicum* CBMAI 1185 to give alcohol 4-hydroxy-3-iodophenylethanol, and it was also oxidized *via* a Baeyer–Villiger reaction to produce the phenylacetate derivative as an intermediate which was hydrolysed yielding 2-iodobenzene-1,4-diol (Rocha et al., 2012c).

*O*-Fluoro-, *o*-chloro- *o*-bromo, and *o*-nitroacetophenones were also bioconverted by marine fungi *Botryosphaeria* sp. Br-09, *Eutypella* sp. Br-023, *Hydropisphaera* sp. Br-27, *Xylaria* sp. Br-61, *Pestalotiopsis* sp. SMA2-C, *Penicillium* sp. SMA2-8 and *Arthopyrenia* sp. SGPY-41. All fungi catalyzed the reduction of *o*-acetophenones with excellent enantiomeric excesses, but yields varied significantly depending on the microorganism and substrate employed. *R* enantiomer was only produced by strains *Pestalotiopsis* sp. SMA2-C and *Penicillium* sp. SMA2-8 with substrate *o*-fluoro, and by *Eutypella* sp. Br-023 and *Hydropisphaera* sp. Br-27 with the compound *o*-bromo (Mouad et al., 2012).

Rocha et al. (2012a) also studied the bioconversion of 1-(4-methoxyphenyl)ethanone. Nine strains of marine-derived fungi were screened but only six were able to catalyze the reduction of this compound with high enantiomeric excesses and excellent yields. *A. sclerotiorum* CBMAI 849, *P. miczynskii* Gc5 and *B. felina* CBMAI 738 reduced it to (*S*)-1-(4-methoxyphenyl)ethanol while *P. citrinum* CBMAI 1186, *Bionectria* sp. Ce5, and *A. sydowii* Ce15 produced (*R*)-1-(4-methoxyphenyl)ethanol. The highest yields were obtained by *A. sclerotiorum* CBMAI 849, *Bionectria* sp. Ce5 and *B. felina* CBMAI 738.

The use of whole living cell immobilization techniques in biocatalytic processes has been increasing in pharmaceutical and food industries. Thus, whole mycelia of the marine fungi *A. sclerotiorum* CBMAI 849 and *P. citrinum* CBMAI 1186, isolated from the marine alga *Caulerpa* sp. and the cnidarian *P. variabilis*, were immobilized on support matrices of silica gel, silica xerogel and/or chitosan for the asymmetric reduction of 1-(4-methoxyphenyl)ethanone and 2-chloro-1-phenylethanone. *P. citrinum* CBMAI 1186 immobilized on chitosan gave the best result quantitatively affording the enantiomer (*S*)-1-(4-methoxyphenyl)ethanol with excellent enantioselectivity. Non-immobilized *P. citrinum* CBMAI 1186 catalyzed the anti-Prelog reduction (enantiomer *R*) with moderate yield and optical purity. *P. citrinum* CBMAI 1186 immobilized on chitosan also catalyzed the reduction of 2-chloro-1-phenylethanone, but without any selectivity. *A. sclerotiorum* CBMAI 849 immobilized on silica gel and the free mycelium catalyzed the bioreduction of 1-(4-methoxyphenyl)ethanone and 2-chloro-1-phenylethanone to alcohols *S-*1-(4-methoxyphenyl)ethanol and *R*-2-chloro-1-phenylethanol, respectively, with excellent results, but when immobilized on silica xerogel did not produce any reduction. These results indicate that the biocatalytic reduction of this type of ketone by immobilization of marine fungi depends on the substrate and the support used (Rocha et al., 2012b).

Liu et al. (2018c) published several studies on the reduction of various substrates by several marine fungi. They examined the synthetic potential of 13 Chinese marine fungi for enantioselective reduction of aromatic ketones. They first optimized biotransformation conditions for the strain *R. mucilageinosa* GIM 2.157 and the best conditions were employed with the rest of the fungi. Only six of them were able to reduce the substrates, especially *Rhodotorula rubra* AS 2.2241 and *R. rubra* GIM 2.31. These fungi reduced ketones to the corresponding alcohols, exhibiting excellent activity and enantioselectivity. In general, when the tested substrates were aromatic ketones without substituents in the benzene ring, the yield and enantiomeric excesses were very high (˃99%). Interestingly, most substrates were reduced to the corresponding (*S*)-alcohols following Prelog’s rule.

The same authors subsequently conducted a comparative study of the asymmetric reduction of ketones. They checked the bioreduction of 1-(3-bromophenyl)ethanone by growing and resting cells of several marine-derived fungi isolated from sediments collected from Guangdong Province, China. Most of the marine-derived fungi catalyzed reduction of the substrate to the corresponding enantiomerically pure (*S*)-alcohols with both methods, but the highest yield and enantiomeric excess were achieved by *R. rubra* AS 2.2241 and therefore this microorganism was chosen for the bioconversion of the rest of the compounds (Liu et al., 2018a).

That same year, Liu et al. (2018b) studied immobilized and free cells of *Geotrichum candidum* for the asymmetric reduction of aromatic ketones. The marine fungus *G. candidum* was isolated from the Chinese cnidarian zoanthid, *P. variabilis*, collected from Zhuhai City. They demonstrated that immobilized *G. candidum* AS 2.361 exhibited better acid–alkaline resistance, higher thermostability, better storage stability and reusability, as well as a lower production cost compared to free cells. Most of the substrates were biotransformed to the corresponding (*S*)-alcohols. (*R*)-enantiomer only was produced from 2-chloro-1-phenylethanone and 2-bromo-1-phenylethanone.

The endophytic fungi *Botryosphaeria* sp. CBMAI 1197, *Eutypella* sp. CBMAI 1196, *Hidropisphaera* sp. CBMAI 1194 and *Xylaria* sp. CBMAI 1195, isolated from the marine red alga *B. radicans*, catalyzed the reduction of fluoroacetophenone derivatives to the corresponding fluorophenyl alcohols. In the biotransformation of 2,2,2-trifluoro-1-phenylethanone, all marine fungi exhibited whole enantioselectivity, giving (*S*)-2,2,2-trifluoro-1-phenylethanol. The fungus *Botryosphaeria* sp. CBMAI 1197 exhibited the best biocatalytic potential leading to the highest conversion values (~100%). This biocatalyst also enantioselectively produced the chiral alcohols *S* and *R*, respectively, from the respective substrates 1-(2-(trifluoromethyl)phenyl)ethanone and 1-(2,4,5-trifluorophenyl)ethanone (Mouad et al., 2015).

Serra et al. (2016) evaluated the reduction of several 1-aryl-2-ethanones with marine yeasts isolated from sediments taken off the coast of New Zealand. Strains *R. mucilaginosa* UBOCC-A-214025 and *R. mucilaginosa* UBOCC-A-214036 rendered the corresponding enantiomerically pure (*S*)-alcohols, with very high enantioselectivity, except for 1-(pyridin-3-yl)ethan-1-one which was not transformed. However, strain *M. guillermondii* UBOCC-A-214008 biotransformed that compound affording the (*S*)-alcohol with good enantioselection and yield, but no activity was found with any of the other substrates.

Hydrogenation of *α*,*β-*unsaturated ketones catalyzed by ene reductases is a common reaction in organic synthesis but control of regioselectivity and chemoselectivity is often very difficult. However, the use of microorganisms as biocatalysts is an interesting alternative to traditional methods. In this connection, Ferreira et al. (2015b) studied the biohydrogenation of *α*,*β*- and *α*,*β*,*γ*,*δ*-unsaturated ketones (**184–189**) by the fungal strain *P. citrinum* CBMAI 1186, isolated from the marine alga *Caulerpa* sp. collected off the coast of the State of São Paulo, Brazil, in a biphasic system of phosphate buffer and *n*-hexane (9,1 proportion was identified as optimal).

**Figure 9:**
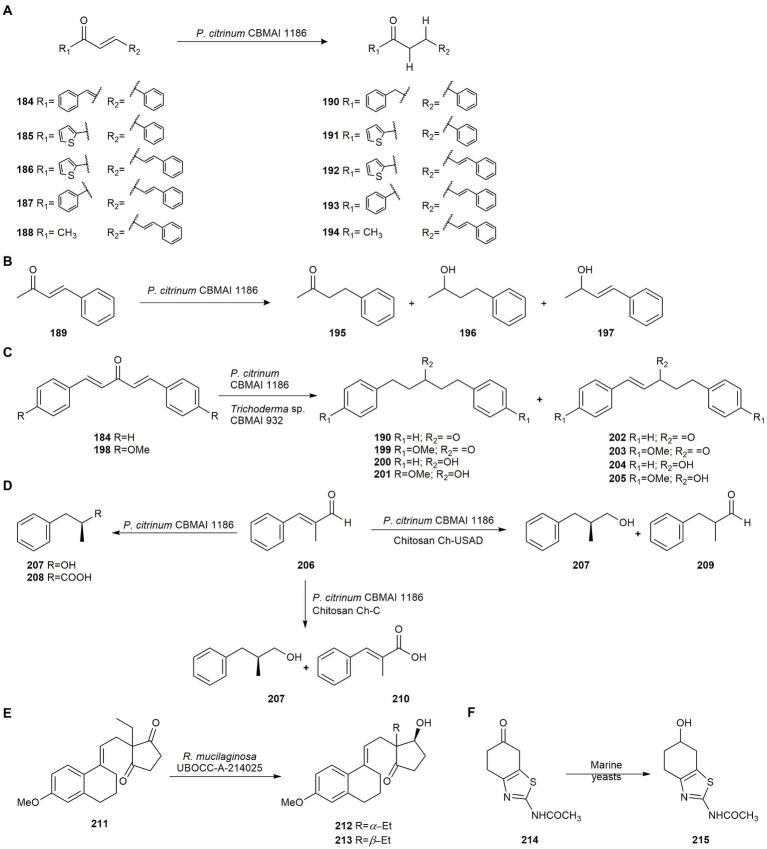
Biotransformations of carbonyl compounds. **(A)** Hydrogenation of the *α*,*β-*unsaturated ketones **184–188** by *Penicillium citrinum* CBMAI 1186 (Ferreira et al., 2015b). **(B)** Reduction of the *α*,*β-*unsaturated ketone **189** by *P. citrinum* CBMAI 1186 (Ferreira et al., 2015b). **(C)** Hydrogenation of bis-*α*,*β-*unsaturated enones **184** and **198** by *P. citrinum* CBMA1186 and *Trichoderma* sp. CBMAI 932 (Ferreira et al., 2015a). **(D)** Biotransformation of (*E*)-2-methyl-3-phenylacrylaldehyde (**206**) by *P. citrinum* CBMA1186 (Ferreira et al., 2020). **(E)** Biotransformation of ethyl secodione (**211**) by *Rhodotorula mucilaginosa* UBOCC-A-214025 (Serra et al., 2016). **(F)** Bioreduction of the ketone **214** by *Meyrozyma guillermondii* and *R. mucilaginosa* UBOCC-A-214025 (Serra et al., 2016).

Biotransformation of the *α*,*β-*unsaturated ketone **184** by *P. citrinum* CBMAI 1186 led to chemoselective bioreduction in preference over the carbonyl group and *γ*,*δ* double bonds with very good yield after 6 days, producing compound **190**. The same results were observed employing monounsaturated ketones **185–188**, obtaining compounds **191–194** at a high conversion (Figure 9A). However, a different result was observed with ketone **189** where the reduction yielded two main products (**195** and **196**) and trace amounts of compound **197** (Figure 9B). It is worth noting that this type of chemo- and regioselective reduction is very hard to achieve using nucleophilic agents from traditional reducing agents, thus *P. citrinum* CBMAI 1186 biohydrogenations may be an interesting alternative in biocatalytic methods (Ferreira et al., 2015b).

In another study, the same authors hydrogenated bis-*α*,*β*-unsaturated enones **184** and **198** using two strains of marine-derived fungi, *P. citrinum* CBMA1186 and *Trichoderma* sp. CBMAI932 affording compounds **190**, **199**–**205** (Figure 9C). The substrates were analogs of the natural product curcumin, a compound whose derivatives exhibit interesting biological activities. Results revealed the presence of oxidoreductases and ene-reductases in the fungal strains (Ferreira et al., 2015a).

Ferreira et al. (2020) also used the strain *P. citrinum* CBMA1186, free and immobilized, on two types of chitosan (Ch-USAD and Ch-C), for the biotransformation of (*E*)-2-methyl-3-phenylacrylaldehyde (**206**), showing different routes in the bioconversion, although in all cases the formation of (*S*)-2-methyl-3-phenylpropan-1-ol (**207**) was observed. When the free mycelium was used, compounds (*S*)-2-methyl-3-phenylpropan-1-ol (**207**) and (*S*)-2-methyl-3-phenylpropanoic acid (**208**) were mostly obtained, whereas for mycelia immobilized on Ch-USAD, the formation of 2-methyl-3-phenylpropan-1-ol (**207**) and 2-methyl-3-phenylpropanal (**209**) was favored. However, for mycelia immobilized on Ch-C, the production of 2-methyl-3-phenylpropan-1-ol (**207**) with 49% yield and (*E*)-2-methyl-3-phenylacrylic acid (**210**) with 35% yield was favored (Figure 9D).

Serra et al. (2016) evaluated the biocatalytic potential in seawater of several strains of the marine yeasts *R. mucilaginosa* and *M. guillermondii* isolated from deep sub-seafloor sediments taken off the coast of New Zealand, for the reduction of key intermediates for the synthesis of compounds of pharmaceutical interest. The biotransformation of ethyl secodione (**211**) provides the key chiral precursor of several hormonal contraceptives. The reduction by *R. mucilaginosa* of the carbonyl group afforded a mixture of diastereoisomers **212** and **213**, but with very high enantioselectivity (Figure 9E). The bioreduction of the ketone **214** by the two marine yeasts was also evaluated rendering the compound 2-acetylamino-6-hydroxy-4,5,6,7-tetrahydrobenzothiazole (**215**; Figure 9F), precursor of the anti-Parkinson (*S*)-pramipexole. Strain *M. guilliermondii* was not enantioselective whereas *R. mucilaginosa* UBOCC-A-214025 afforded the highest *ee*.

## 5. Biotransformation of miscellaneous compounds

Glycidyl ether derivatives are considered as potentially useful intermediates in the synthesis of *β*-adrenergic blockers. This chemical synthesis process is often hampered because of the generation of racemic products and therefore novel alternatives in asymmetric synthesis are needed. In this connection, whole cells of four marine-derived fungi strains, *A. sydowii* Gc12, *P. raistrickii* Ce16, *P. miczynskii* Gc5 and *Trichoderma* sp. Gc1, were evaluated by Martins et al. (2011) for the enzymatic resolution of racemic (±)-2-(benzyloxymethyl) oxirane (**216**).

**Figure 10:**
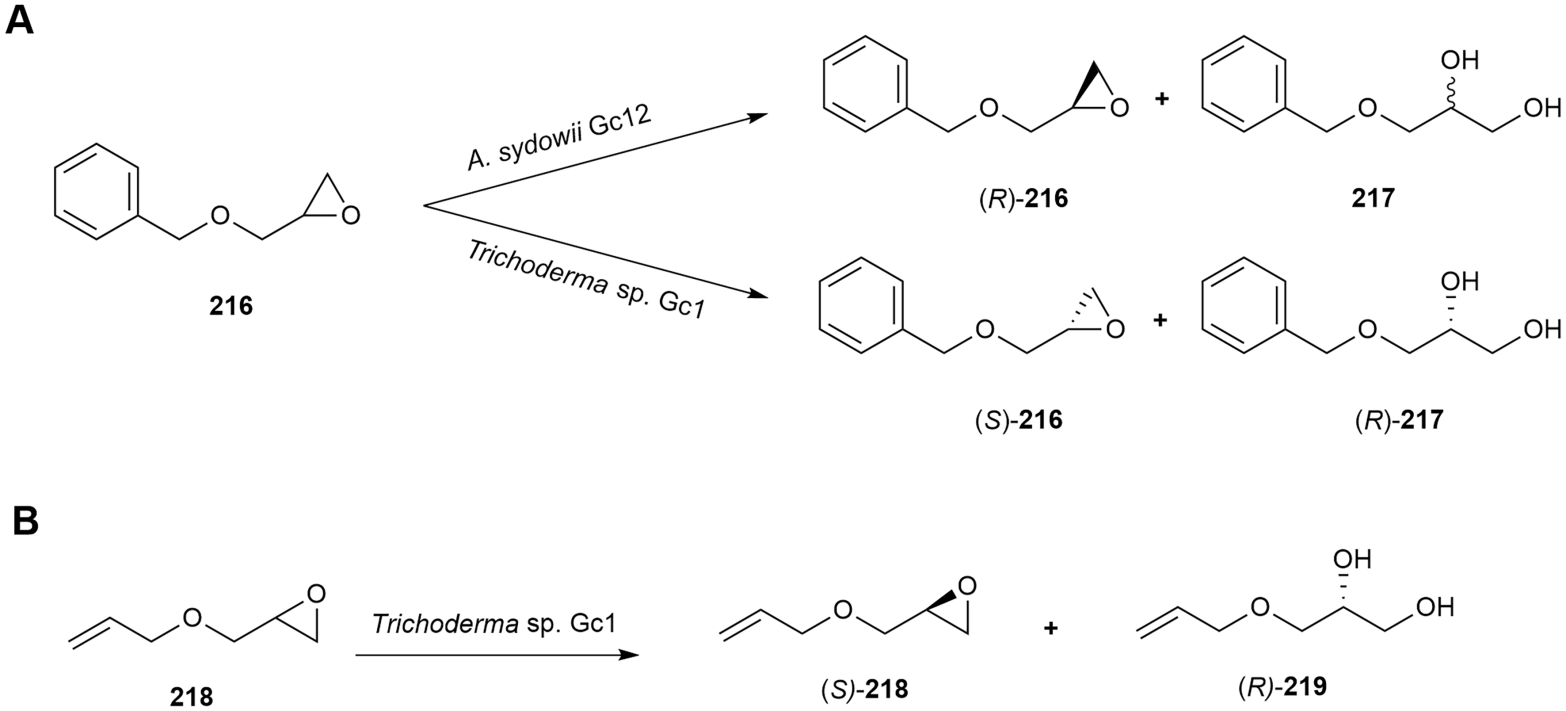
Biotransformations of miscellaneous compounds. **(A)** Enzymatic resolution of racemic (±)-2-(benzyloxymethyl) oxirane (**216**) by *Aspergillus sydowii* Gc12 and *Trichoderma* sp. Gc1 (Martins et al., 2011). **(B)** Hydrolysis of (±)-2-(allyloxymethyl) oxirane (**218**) by *Trichoderma* sp. Gc1 (Martins et al., 2012).

Although *P. raistrickii* Ce16 and *P. miczynskii* Gc5 did not biocatalyse the xenobiotic substrate, the other two strains exhibited regioselective hydrolytic activity. *A. sydowii* Gc12 catalyzed the epoxide group hydrolysis of compound **216** with a good conversion rate, producing (*R*)-**216** with an enantiomeric excess of 46% and diol **217**, with a poor enantiomeric excess of 10% (absolute configuration not determined). *Trichoderma* sp. Gc1 exhibited complementary stereoselectivity in opening the epoxide ring of **216** presenting an (*R*)-enantiomer preference, yielding (*S*)-**216** with good enantiomeric excess of 60% and diol (*R*)-**217**, with an enantiomeric excess of 32% due to a *β* attack at the terminal carbon (Figure 10A; Martins et al., 2011).

Hydrolysis of (±)-2-(allyloxymethyl) oxirane (**218**) by whole cells of the marine fungus *Trichoderma* sp. Gc1 mostly produced (*S*)-(+)-2-(allyloxymethyl)oxirane ((*S*)-**218**; 34% *ee*) and (*R*)-(−)-3-(allyloxy)propane-1,2-diol ((*R*)-**219**; 10% *ee*; Figure 10B). Results showed that the fungal hydrolases exhibited selectivity with preference for oxirane *R*, while the formation of (*R*)-diol **219** indicated retention of configuration as the mechanism involved (Martins et al., 2012).

## 6. Biodegradation

Environmental pollution has been on the rise in the past few decades owing to unsafe agricultural practices, rapid industrialization, and the need to generate cheap forms of energy. This increased human activity has led to the continuous release of highly toxic and recalcitrant organic chemicals into the biosphere, such as plastics, petroleum derivatives, pesticides, etc.

Several methods have been tried to remove pollutants from the environment, such as incineration, landfilling and dredging, or a combination of these (Nikolaivits et al., 2021). However, these conventional methods are expensive, time-consuming and can release toxic compounds or produce secondary pollutants. A more cost-effective and eco-friendly alternative is bioremediation which involves harnessing the diverse metabolic capabilities of microorganisms to biotransform contaminants into harmless products (Arun et al., 2008). As already mentioned, marine-derived microorganisms possess a unique enzyme system and can thrive under extreme conditions, which makes them excellent candidates for bioremediation applications.

### 6.1. Petroleum hydrocarbons

Petroleum hydrocarbons (PHCs) are one of the most widespread and heterogeneous organic pollutants affecting marine ecosystems. They are mainly composed of alkanes, olefins and aromatics. Due to their poor solubility in water, PHCs are readily adsorbed onto particles and settle to sea-bottom sediments where they persist for long periods of time causing a significant negative impact on benthic aquatic communities (Dell’ Anno et al., 2021).

This type of pollution has received enormous attention due to its toxicity, persistence and accumulation in aquatic habitats. Bioremediation approaches based on the use of microorganisms for marine sediment remediation are gaining increasing attention due to their ecological nature and lower cost.

Deshpande’s group studied the ability of the yeast *Yarrowia lipolytica* NCIM 3589 (current name *Y. lipolytica* var. *indica*), isolated from oil-contaminated seawater near Mumbai (India), to degrade pure alkanes. *Y. lipolytica* is a dimorphic strain which has the capacity to change its morphology from yeast to mycelium in response to environmental and nutritional conditions. In yeast form, this strain was able to degrade 20–60% of the pure *n*-alkanes added (10, 12, 14, 16, 18, and 20 carbon atoms) within 48 h under aerobic conditions, with *n*-hexadecane being the most degraded alkane (60%). This hydrocarbon was more efficiently metabolized by the yeast cells of var. *indica* than by other terrestrial strains. Mycelial cells of *Y. lipolytica* NCYC 3589, produced under low oxygen concentrations, degraded alkanes only after reverting to the yeast cells (Zinjarde et al., 1998; Palande et al., 2014; Campos-Góngora et al., 2018).

Phenylalkanes are persistent organic pollutants which are mutagenic and carcinogenic. Nhi-Cong et al. (2016) reported the biodegradation of *sec*-hexylbenzene by the yeast *Trichosporon asahii* B1, isolated from oil-contaminated sediments collected from the coastal area of Quangninh in Vietnam. This marine yeast transformed this branched side-chain benzene with biofilm-forming cells more efficiently than with planktonic cells. The compounds involved in its bioconversion were 5-phenylhexanoic acid, 3-phenylbutyric acid, 2-phenylpropionic acid, *β*-methylcinnamic acid, acetophenone, benzoic acid, and 2,3-dihydroxybenzoic acid. The identification of numerous phenylalkanoic acids containing shorter branched side chains and/or shorter unsaturated side chains than *sec*-hexylbenzene as biotransformation products suggests that the degradation of this branched aromatic hydrocarbon started mainly at the side chain.

Polycyclic aromatic hydrocarbons (PAHs) are widely distributed contaminants with adverse effects on both humans and the environment. Due to their persistence, toxicity, mutagenicity and carcinogenicity, PAHs have caused significant environmental concern. Although these pollutants may undergo adsorption, volatilization, photolysis, and chemical degradation, microbial degradation is the major degradation process. Microbial degradation biotransforms PAHs into less complex metabolites and, through mineralization, into inorganic minerals, H_2_O, CO_2_ under aerobic and CH_4_ under anaerobic conditions (Haritash and Kaushik, 2009; Premnath et al., 2021).

Several marine-derived fungi capable of metabolizing PAHs have been described. They have been isolated from various marine environments such as seawater, sediments, marine organisms, marshes and estuaries (Nikolaivits et al., 2017). Their use in the bioremediation of contaminated saline environments is facilitated by their tolerance of saline conditions.

Sette’s group examined several marine-derived fungi isolated from different Brazilian cnidarians and sponges for their ability to biodegrade pyrene and benzo[a]pyrene. The filamentous fungus *Aspergillus sclerotiorum* CBMAI 849, isolated from the scleractinian coral *M. hispida*, showed excellent degradation of pyrene (99.7%) and benzo[a]pyrene (76.6%) after 8 and 16 days, respectively. Substantial benzo[a]pyrene depletion was also achieved after 16 days of incubation with the zygomycete *M. racemosus* CBMAI 847 (51.7%), isolated from the zoanthid *P. variabilis*, and by the hyphomycete *Cladosporium cladosporioides* CBMAI 857 (45.3%), isolated from *P. caribaeorum*. CBMAI 849 and CBMAI 847 strains metabolized pyrene and benzo[a]pyrene to pyrenylsulfate and benzo[a]pyrenylsulfate, respectively, which are less toxic than their respective parent compounds. In addition, hydroxypyrene and hydroxybenzo[a]pyrene were detected as intermediates, suggesting that the degradation mechanism of PAHs involves hydroxylation mediated by a cytochrome P-450 monooxygenase, followed by conjugation with sulfate ions (Passarini et al., 2011). The conjugation reaction with sulfate or nitrate is considered as a detoxification action by microorganisms to protect themselves from the higher toxicity of the free hydroxylated metabolites (Lambert et al., 1995; Capotorti et al., 2005).

In another study, they screened three marine-derived basidiomycete fungi isolated from the Brazilian sponges *D. reticulatum* and *Amphimedon viridis*. *Marasmiellus* sp. CBMAI 1062 was able to degrade 97.2% of benzo[a]pyrene after 7 days of incubation and 98.2% of pyrene after only 48 h of incubation under saline conditions. Lower detoxification of both PAHs was observed after 21 days of incubation with *Tinctoporellus* sp. (almost 50%) and *Peniophora* sp. (30%). The CBMAI 1062 strain metabolized pyrene to hydroxypyrene, pyrene dihydrodiol, and dihydroxypyrene. These compounds were identified by GC–MS and therefore the position of the hydroxyl groups could not be determined. The intermediate metabolites generated suggest that the fungus degraded pyrene *via* the cytochrome P450 system and epoxide hydrolases. Toxicity tests using *Artemia* sp. showed a loss of toxicity after pyrene degradation by CBMAI 1062 (95% of survivors with active motility vs. 67.2% of survivors in abiotic control), highlighting the biotechnological potential of this fungus to detoxify this environmental pollutant (Vieira et al., 2018).

**Figure 11:**
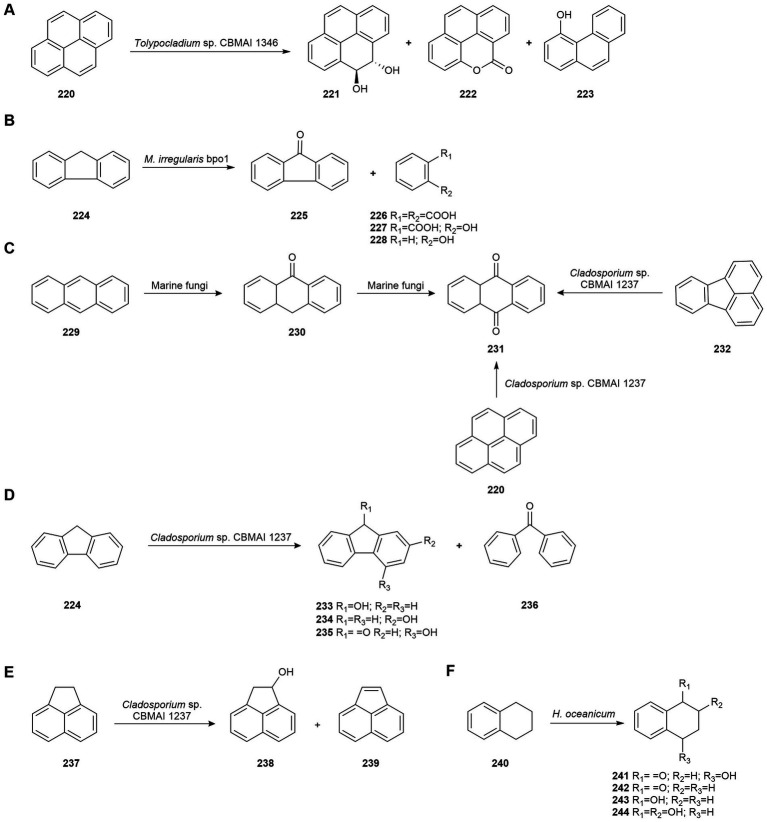
Biodegradations of petroleum hydrocarbons. **(A)** Pyrene (**220**) biodegradation by *Tolypocladium* sp. CBMAI 1346 (Vasconcelos et al., 2019). **(B)** Fluorene (**224**) biodegradation by *Mucor irregularis* bpo1 (Bankole et al., 2021b). **(C)** Biodegradation of anthracene (**229**), anthrone (**230**), pyrene (**220**), and fluoranthene (**232**) by *Cladosporium* sp. CBMAI 1237 (Birolli et al., 2018a). **(D)** Fluorene (**224**) biodegradation by *Cladosporium* sp. CBMAI 1237 (Birolli et al., 2018a). **(E)** Acenaphthene (**237**) biodegradation by *Cladosporium* sp. CBMAI 1237 (Birolli et al., 2018a). **(F)** 1,2,3,4-Tetrahydronaphthalene (**240**) biodegradation by *Hypoxylon oceanicum* (Li et al., 2005).

Recently, Sette’s group reported that the fungus *Tolypocladium* sp. CBMAI 1346, isolated from marine sponges collected in Brazil, reduced pyrene (**220**) by 64.43% under non-saline conditions and 95.54% in optimized saline conditions after 7 days of incubation. The pattern of pyrene degradation metabolites produced by this strain was very different in the two culture conditions. The non-optimized process gave rise to compounds **221**–**223** (Figure 11A), while the optimized process generated only metabolites with aliphatic chains (hexadecane, icos-1-ene, icos-5-ene, octadec-1-ene, docosan-1-ol, and hexadec-1-ene) suggesting that under these conditions the metabolization of pyrene (**220**) by the fungus may have been accelerated. The metabolic intermediates generated and transcriptomic data revealed that degradation occurred mainly *via* the cytochrome P450 pathway and auxiliary enzymes (e.g., phenol 2-monooxygenase, epoxide hydrolases, and some dioxygenases). The optimized process was able to degrade pyrene without the generation of toxic compounds after 21 days using *Artemia* sp. as a bioindicator (Vasconcelos et al., 2019).

Wu et al. (2009) evaluated the fungus *Aspergillus* sp. BAP14 isolated from marine sediments collected off the coast of Xiamen City (China) for its ability to biodegrade benzo[a]pyrene. This strain was able to remove about 30 and 60% of the compound after 3 and 12 days of incubation, respectively, employing an initial concentration of 10 mg L^−1^. The addition of naphthalene significantly enhanced removal percentages of benzo[a]pyrene.

Fluorene (**224**) is a concerning environmental compound because of its carcinogenicity, teratogenicity, mutagenicity, toxicity and resistance to microbial degradation. Bankole et al. (2021b) studied its biodegradation by the marine-derived fungus *Mucor irregularis* bpo1, isolated from soil collected from the Atlantic Ocean shore of Nigeria, by Response Surface Methodology (RSM) using Box–Behnken Design (BBD) and Central Composite Design (CCD). Thanks to this strategy, a maximum biodegradation rate of 81.5% of fluorene (**224**) was achieved in 5 days. Glucose and manganese ions were found to significantly improve the biodegradation of this aromatic hydrocarbon. Moreover, four metabolites were identified as biodegradation products: 9*H*-fluoren-9-one (**225**), benzene-1,2-dicarboxylic acid (**226**), 2-hydroxybenzoic acid (**227**), and phenol (**228**; Figure 11B). The enzyme activities revealed 87, 59 and 31% induction of laccase, manganese peroxidase and lignin peroxidase, respectively. The efficiency exhibited by the marine-derived fungus *M. irregularis* bpo1 makes it a promising alternative to fluorene bioremediation.

Similarly, the biodegradation of chrysene by *C. lunatus* strain CHR4D, a marine-derived ascomycete fungus isolated from crude oil-contaminated sediment collected from the Alang-Sosiya ship-breaking yard in India, was optimized by RSM using CCD. The two-step optimization protocol led to a high biodegradation rate of 93.1% on the fourth day versus 56.4% on the fourteenth day using non-optimized conditions (Bhatt et al., 2014).

The lignin-degrading marine fungus *Flavodon flavus*, strain NIOCC # 312, isolated from decomposing seagrass leaves of *Thalassia hemprichii* (Ehrenberg) Ascherson, was able to remove about 70–80% of phenanthrene and 71–78% of chrysene after 6 days, when manganese peroxidase production was maximum. According to the studies conducted phenanthrene is instantly adsorbed by the fungal biomass and subsequently degraded by lignin-degrading enzymes present in the cell wall and in the exopolymeric matrix around the fungal hyphae (Raghukumar et al., 2006).

Birolli et al. (2018a) examined the capability of fungi isolated from marine sponges obtained at a non-contaminated site off the coast of São Sebastião (Brazil) to biodegrade anthracene (**229**). *T. harzianum* CBMAI 1677*, Cladosporium* sp. CBMAI 1237*, A. sydowii* CBMAI 935*, P. citrinum* CBMAI 1186, and *M. racemosus* CBMAI 847 were able to biodegrade anthracene (**229**) at different rates. However, *Cladosporium* sp. CBMAI 1237 was the most efficient strain and after 21 days biodegraded 71% of anthracene (**229**) to anthrone (**230**) and subsequently to anthraquinone (**231**; Figure 11C). Biodegradation was higher in the presence of artificial seawater (42% biodegradation after 14 days compared to 26% in its absence), suggesting that the biodegradation of PAHs may be faster in seawater than in non-saline environments.

The most efficient strain, *Cladosporium* sp. CBMAI 1237, was selected for the degradation of other PAHs. After 21 days, it metabolized 100% of anthrone (**230**), 62% of pyrene (**220**), and 52% of fluoranthene (**232**) to anthraquinone (**231**; Figure 11C). Anthraquinone and phenanthrene were also degraded (32 and 47%, respectively), but no biotransformation products were detected. CBMAI 1237 strain also biodegraded 70% of fluorene (**224**) to 9-hydroxyfluorene (**233**), 2-hydroxyfluorene (**234**), 4-hydroxy-9-fluorenone (**235**), and benzophenone (**236**; Figure 11D), and 78% of acenaphthene (**237**) to acenaphthen-1-ol (**238**) and acenaphthylene (**239**; Figure 11E). Aminopyrene, hydroxypyrene and anthraquinone (**231**) were identified as biotransformation products of nitropyrene by CBMAI 1237. Therefore, anthraquinone (**231**) was a common biodegradation product for several PAHs (Birolli et al., 2018a).

Álvarez-Barragán et al. (2021) examined the tolerance and ability of 85 fungal strains from coastal sediments contaminated with PAHs to biodegrade this type of hydrocarbons. *Alternaria destruens* F10.81 performed the best with more than 80% removal in the case of phenanthrene, pyrene (**220**) and fluoranthene (**232**), and about 65% for benzo[a]pyrene. These studies did not determine whether the PAH-removal potential of the isolated strains involves biosorption and/or monooxygenase (i.e., cytochrome P450) degradation mechanisms.

Similarly, the ability of pelagic sediment isolates from different locations in the two gulfs of Gujarat and from the Arabian sea to tolerate and degrade PAHs was evaluated. Based on the GC–MS profile, *Penicillium ilerdanum* NPDF1239-K3-F21 and *Aspergillus versicolor* NPDF190-C1-26 exhibited efficiency rates of >75% in degrading naphthalene, phenanthrene, pyrene (**220**), anthracene (**229**), and fluoranthene (**232**; Mahajan et al., 2021).

Li et al. (2005) examined the biotransformation of 1,2,3,4-tetrahydronaphthalene (**240**) by the marine fungus *Hypoxylon oceanicum* from the South China Sea. This fungus biotransformed the naphthalene derivative mainly to 4-hydroxy-3,4-dihydronaphthalen-1(2*H*)-one (**241**; 96.16%) and to three minor products, 3,4-dihydronaphthalen-1(2*H*)-one (**242**), 1,2,3,4-tetrahydronaphthalen-1-ol (**243**), and 1,2,3,4-tetrahydronaphthalene-1,2-diol (**244**; Figure 11F). Metabolic intermediates obtained revealed that the biodegradation of 1,2,3,4-tetrahydronaphthalene (**240**) occurred though oxidation reactions on the non-activated alicyclic skeleton.

### 6.2. Pesticides

Pesticides, despite having a beneficial effect on agricultural production, may be one of the most dangerous contaminants to the environment since they are very toxic and have adverse effects on human and animal health. They can also bioaccumulate, remaining in the soil at the application site, or may be transported to different parts of the environment such as sediments, plants, surface and ground waters, marine environments and even volatilized into the atmosphere, depending on their physical–chemical properties (Vacondio et al., 2015b). Among the techniques employed to remove these contaminants, bioremediation emerges as a safe, low-cost, and environmentally friendly alternative technology.

Pesticides may be classified as organochlorines, organophosphates, carbamates, and pyrethroids. Organochlorines cause great concern due to their high toxicity and resistance to degradation. Pentachlorophenol is a phenolic organochlorine used as a pesticide, disinfectant and food preservative, the use of which has been banned in most countries due to its high toxicity and slow biodegradation. Vacondio et al. (2015a) examined its bioremediation by marine-derived fungi isolated from ascidian *Didemnum ligulum* collected in São Sebastião (Brazil).

Of all the fungi tested, *T. harzianum* CBMAI 1677 was the strain most resistant to high concentrations of pentachlorophenol and was therefore selected for pesticide biodegradation. After 7 days, this pesticide was biotransformed into pentachloroanisole by biomethylation, which was subsequently dechlorinated to 2,3,4,6-tetrachloroanisole. Their biodegradation products were also partially metabolized by this strain, preventing their accumulation in the environment (Vacondio et al., 2015b).

2,4-Dichlorophenol has been widely used as a pesticide, fungicide and wood preservative. As it has been released into the environment in large quantities, it has been classified as a priority pollutant together with other chlorophenols. Nikolaivits et al. (2019, 2020) screened 87 fungal strains isolated from corals, sponges, tunicates, and bivalves from the Mediterranean Sea, Red Sea, and Adaman Sea, for their ability to biodegrade 2,4-dichlorophenol at a concentration of 1 mM. While most of the strains removed the pesticide, only 9 were able to reach bioconversion yields of over 55%. *Chrysosporium* sp. TM9-S2 was the most effective strain with a removal rate of 74.0%, *Aspergillus* sp. TM124-S1 removed 69.0%, *Tritirachium* sp. ML197-S3 66.3%, *Cladosporium* sp. ML6-S1 64.0%, *Aspergillus creber* TM122-S3 62.0%, *Penicillium chrysogenum* ML156-S8 59.5%, *Penicillium steckii* TM2-S5 58.5%, *Penicillium* sp. TM38-S1 56.2%, and *Aspergillus* sp. ML147-S2 55.1%. These strains biodegraded 2,4-dichlorophenol to hydroxylated derivatives, oxidative or reductive dechlorination products, and sulphated, palmitate, glucoside, glutamine and cysteine conjugates, all detected by UHPLC-HRMS/MS analysis. Strains TM122-S3, TM124-S1, ML6-S1, ML147-S2, ML156-S8, and ML197-S3 managed to oxidatively dechlorinate the starting compound, which is very important as this substantially decreases its toxicity. However, only strain ML197-S3 expressed high levels of extracellular catechol 1,2-dioxygenase activity and produced a cleavage of the aromatic structure of the starting compound after dechlorination, indicating the assimilation of the xenobiotic by the fungus.

Another of the most extensively used organochlorine pesticides in the past was dieldrin, whose high toxicity and long persistence in the environment led to its ban more than 50 years ago. However, it is still present in many ecosystems, posing a serious problem for the environment. To biodegrade this pesticide, Birolli et al. (2015) evaluated the marine-derived fungi *A. sydowii* CBMAI 935, *A. sydowii* CBMAI 933, *P. miczynskii* CBMAI 930, and *Trichoderma* sp. CBMAI 932 isolated from the sponges *G. corticostylifera* and *C. erecta*. All fungal strains were able to grow in the presence of the pesticide, but *P. miczynskii* CBMAI 930 exhibited the highest tolerance. This strain managed to degrade 90% of dieldrin after 14 days in the presence of hydrogen peroxide used to promote peroxidases activity. No biodegradation products were identified by GC–MS analyses, suggesting that dieldrin may be mineralized or transformed into polar compounds by conjugation reactions, preventing the appearance of toxic or long-lasting derivatives.

The ability of these marine fungi to biodegrade the chlorinated insecticide dichlorodiphenylchloroethane (DDD), whose use was banned in the 1970s, was also assessed. DDD is still present in several environments, not only because of its direct use in the past, but also because it is rendered as a breakdown product of the widely used pesticide dichlorodiphenyltrichloroethane (DDT). Among all the strains tested, *Trichoderma* sp. CBMAI 932 (formerly *Trichoderma* sp. Gc1) was the most tolerant to the pesticide. It degraded 58% of DDD after 14 days when 5.0 mg of DDD was added to a 5 day-old culture of *Trichoderma* sp. CBMAI 932 in the presence of hydrogen peroxide. As in the case of dieldrin, no DDD degradation intermediates were detected by GC–MS analysis. However, the enhanced biodegradation of the pesticide in the presence of H_2_O_2_ suggests the involvement of peroxidases (Ortega et al., 2011).

Pyrethroid pesticides are remarkably effective due to improvements over decades of research, making them the third best-selling chemical class of insecticides. The pyrethroid pesticide esfenvalerate (*S*,*S*-fenvalerate, **245**) accumulates in aquatic sediments and is very toxic to aquatic species, so its biodegradation in this ecosystem is very important. Birolli et al. (2014, 2016) examined seven marine-derived fungi as bioremediation agents of esfenvalerate (**245**), *P. raistrickii* CBMAI 931, *A. sydowii* CBMAI 935, *Cladosporium* sp. CBMAI 1237, *Microsphaeropsis* sp. CBMAI 1675, *Acremonium* sp. CBMAI 1676, *Westerdykella* sp. CBMAI 1679, and *Cladosporium* sp. CBMAI 1678, all from the sponges *C. erecta* and *D. reticulatum* collected from a non-contaminated site on the northern coast of São Paulo state in Brazil.

**Figure 12:**
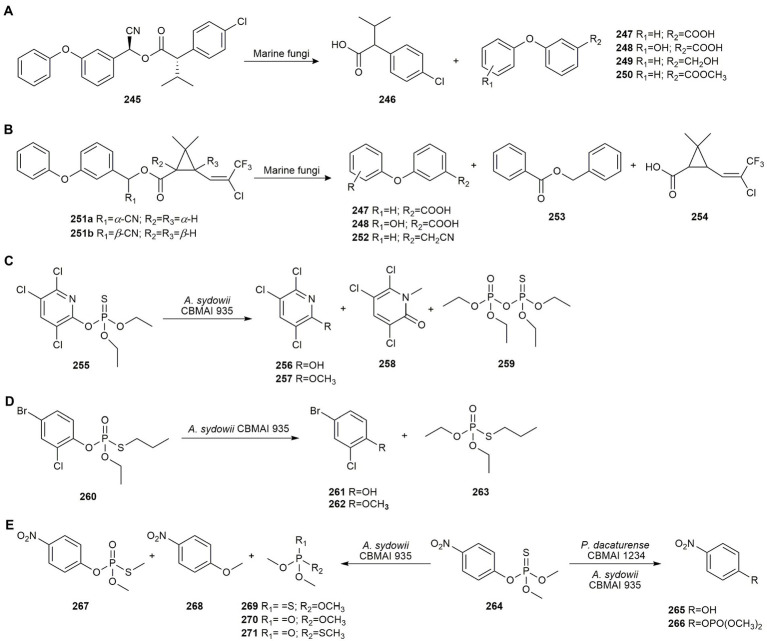
Pesticide biodegradations. **(A)** Esfenvalerate (**245**) biodegradation by marine fungi (Birolli et al., 2016) **(B)** Biodegradation of (±)-lambda-cyhalothrin (**251**) enantiomers by *Aspergillus* sp. CBMAI 1829, *Acremonium* sp. CBMAI 1676, *Microsphaeropsis* sp. CBMAI 1675, and *Westerdykella* sp. CBMAI 1679 (Birolli et al., 2018b). **(C)** Chlorpyrifos (**255**) biodegradation by *Aspergillus sydowii* CBMAI 935 (Soares et al., 2021). **(D)** Profenofos (**260**) biodegradation by *A. sydowii* CBMAI 935 (Soares et al., 2021). **(E)** Bioremediation of methyl parathion (**264**) by *Penicillium decaturense* CBMAI 1234 and *A. sydowii* CBMAI 935 (Alvarenga et al., 2014; Soares et al., 2021).

All the fungal strains were able to biodegrade the pesticide, although CBMAI 1675, CBMAI 1676, and CBMAI 1679 were the most efficient. Esfenvalerate (**245**) was metabolized to 2-(4-chlorophenyl)-3-methylbutyric acid (**246**), 3-phenoxybenzoic acid (**247**), and its hidroxylated derivative **248** by all the strains. In addition, *Microsphaeropsis* sp. CBMAI 1675 and *Cladosporium* sp. CBMAI 1678 produced 3-phenoxybenzyl alcohol (**249**) and methyl 3-phenoxybenzoate (**250**), respectively (Figure 12A). Based on the HPLC-ToF results, a biodegradation pathway involving carboxylesterases, oxynitrilases and aldehyde dehydrogenases was proposed (Birolli et al., 2016).

(±)-Lambda-cyhalothrin (**251**) is another pyrethroid insecticide composed of a 1:1 mixture of the enantiomers (1*R*,3*R*,*αS*; **251a**) and (l*S*,3*S*,*αR*; **251b**), where the enantiomer commercially known as gamma-cyhalothrin (**251a**) is the most active. This pesticide is relatively persistent in the environment, so Birolli et al. (2018b) studied its biodegradation by the marine-derived fungi *Aspergillus* sp. CBMAI 1829, *Acremonium* sp. CBMAI 1676, *Microsphaeropsis* sp. CBMAI 1675, and *Westerdykella* sp. CBMAI 1679, isolated from the ascidian *D. ligulum* and the sponge *D. reticulatum*.

All fungal strains biodegraded this insecticide (**251**) to 3-phenoxybenzoic acid (**247**), 3-(hydroxyphenoxy)benzoic acid (**248**), 2-(3-phenoxyphenyl)acetonitrile (**252**), benzyl benzoate (**253**), and (*E*)-3-(2-chloro-3,3,3-trifluoroprop-1-en-1-yl)-2,2-dimethylcyclopropanecarboxylic acid (**254**; Figure 12B). *Aspergillus* sp. CBMAI 1829 was the most efficient strain with a 45% removal rate. Furthermore, all the strains evaluated enantioselectively biodegraded the **251a** enantiomer, indicating that the use of the most active enantiomer as an insecticide not only enables the use of a lower amount of pesticide, but also a more easily biodegradable product thus reducing the risk of environmental contamination (Birolli et al., 2018b).

Organophosphate pesticides are an attractive alternative to organochlorines given their low cost, easy synthesis, increased biodegradability and limited accumulation in living organisms compared to organochlorine pesticides (dos Santos et al., 2007). They are currently one of the most widely used classes of pesticides worldwide. However, they are also highly toxic as they are potent irreversible inhibitors of acetylcholinesterase (AChE), an enzyme that has a profound effect on the nervous system of organisms exposed to it (Edwards and Tchounwou, 2005).

One of the most widely used organophosphate pesticides is chlorpyrifos (**255**), which is hydrolysed to 3,5,6-trichloropyridin-2-ol (**256**) in the environment. This derivative, although it cannot act as an inhibitor of the AChE enzyme, is still a polluting compound. Alvarenga et al. (2015) studied the biodegradation of a commercial formulation of this pesticide (Lorsban 480 BR) and its hydrolysis product by the marine-derived fungi *A. sydowii* CBMAI 935 and *Trichoderma* sp. CBMAI 932, isolated from the sponges *C. erecta* and *G. corticostylifera*, respectively. Both strains, CBMAI 935 and CBMAI 932, degraded on average 63 and 72% of chlorpyrifos (**255**), respectively, and reduced the concentration of **256** after 30 days.

*A. sydowii* CBMAI 935 metabolized chlorpyrifos (**255**) to 3,5,6-trichloropyridin-2-ol (**256**), 2,3,5-trichloro-6-methoxypyridine (**257**), 3,5,6-trichloro-1-methylpyridin-2(1*H*)-one (**258**), and tetraethyl dithiodiphosphate (**259**; Figure 12C). The biotransformation product **259** was produced by a condensation reaction between two *O*,*O*-diethyl phosphorothioates, while the metabolites **256** and **257** were obtained by hydrolysis of the O-P bond by phosphoesterases and subsequent methylation by methyltransferases (Soares et al., 2021).

Profenofos (**260**) is another organophosphate pesticide widely used as a non-systemic foliar insecticide and acaricide. It is classified as moderately hazardous and therefore its bioremediation by marine fungi has been studied. *P. raistrickii* CBMAI 931 and *A. sydowii* CBMAI 935 biodegraded on average 97 and 72% of profenofos (**260**), respectively, after 30 days. In addition, its hydrolysis product, 4-bromo-2-chlorophenol (**261**), was almost completely metabolized by these two strains (da Silva et al., 2013). The metabolites of profenofos (**260**) biodegradation by *A. sydowii* CBMAI 935 were 4-bromo-2-chlorophenol (**261**), 4-bromo-2-chloro-1-methoxybenzene (**262**), and *O*,*O*-diethyl *S*-propylphosphorothioate (**263**). Compound **263** resulted from an ethylation reaction in *O*-ethyl *S*-propyl *O*-hydrogen phosphorothioate (Figure 12D; Soares et al., 2021).

Methyl parathion (**264**), another organophosphate insecticide and acaricide, is widely used for pest control on a wide variety of crops due to its high efficiency. However, it is extremely toxic which is why considerable attention has been given to its degradation and removal from the environment. Alvarenga et al. (2014) studied the ability of the marine-derived fungi *A. sydowii* CBMAI 935 and *P. decaturense* CBMAI 1234 to biodegrade this pesticide. Both strains achieved complete degradation of methyl parathion (**264**) after 20 and 30 days, respectively, and were able to degrade 51 and 40%, respectively of its hydrolysis product *p*-nitrophenol (**265**; Figure 12E).

*P. decaturense* CBMAI 1234 biodegraded the insecticide to its more toxic form methyl paraoxon (**266**), which was subsequently hydrolysed to *p*-nitrophenol (**265**; Alvarenga et al., 2014). *A. sydowii* CBMI 935 metabolized methyl parathion (**264**) to its more toxic oxidation and isomerisation products methyl paraoxon (**266**) and isoparathion (**267**), respectively. These compounds were subsequently biodegraded to the less toxic hydrolysis products *p*-nitrophenol (**265**), 1-methoxy-4-nitrobenzene (**268**), *O*,*O*,*O*-trimethyl phosphorothioate (**269**), trimethyl phosphate (**270**), and *O*,*O*,*S*-trimethyl phosphorothioate (**271**). These results suggest that both strains were efficient for the bioremediation of pesticide (**264**) and its more toxic forms (**266**, **267**; Figure 12E; Soares et al., 2021).

The marine-derived fungi *P. citrinum* DL4M3, *P. citrinum* DL9M3 and *Fusarium proliferatum* DL11A, isolated from ascidian *D. ligulum*, were able to completely degrade methyl parathion (**264**) within 20 days. However, there was no significant difference with the removal of the pesticide by chemical hydrolysis and it could not be confirmed that methyl parathion (**264**) was degraded by fungal action. In contrast, its toxic hydrolysis product *p*-nitrophenol (**265**) was biodegraded at a rate of 90% by *F. proliferatum* DL11A in 30 days (Rodrigues et al., 2016).

### 6.3. Synthetic dyes

Synthetic dyes are mainly used in pulp and paper mills, textile, leather, pharmaceutical and food industries which produce and discharge highly colored effluents. These dye-laden wastewaters, when mixed with large bodies of water, weaken primary productivity, hinder the diffusion of gases and affect human health, besides producing esthetically unacceptable coloration (Vala et al., 2018). In aquatic ecosystems they block the passage of light to lower depths, preventing photosynthesis and causing anaerobic conditions which, in turn, leads to the death of aquatic life and toxic and foul-smelling water (Raghukumar et al., 2008). Therefore, dye waste degradation is a major environmental concern. Since chemical remediation contributes to secondary pollution, an alternative remediation method is needed, biodegradation (mineralization or biotransformation) and adsorption onto biomass being the main means of dye removal (Vala et al., 2018).

Several marine-derived fungi have been reported to successfully degrade synthetic dyes with basidiomycetous fungi leading the list. For example, the basidiomycete fungus *Cerrena unicolor* (NIOCC #2a), isolated from decaying wood collected from mangroves in India, was able to completely decolorize brilliant green and almost completely decolorize reactive aniline blue and Congo red after 4 days, while it was less successful with reactive orange 176. Laccase was the most dominant lignin-degrading enzyme produced by this fungus with very low activities of manganese-dependent peroxidase and no lignin peroxidase activity. The synthetic dyes acted as laccase inducers suggesting the involvement of this enzyme in the bioremediation of these pollutants (D’Souza et al., 2006).

Similarly, the marine-derived fungi *Phialophora* sp. (MF 6) and *Penicillium* sp. (MF 49), isolated from seawater from Manila Bay, and *Cladosporium* sp. (EME 14), isolated from living seagrass collected from Calatagan Bay, completely decolorized 0.01% Congo red. In contrast, only strains EMF 14 and MF 49 decolorized 87 and 91%, respectively of 0.01% crystal violet (Torres et al., 2011). Also, the marine-derived fungus *Aspergillus flavus*, isolated from seawater collected in the Bay of Bengal, was able to remove between 80 and 90% of crystal violet, malachite green, methylene blue B, and safranin dyes in 3 to 7 days (Lalitha et al., 2011).

**Figure 13:**
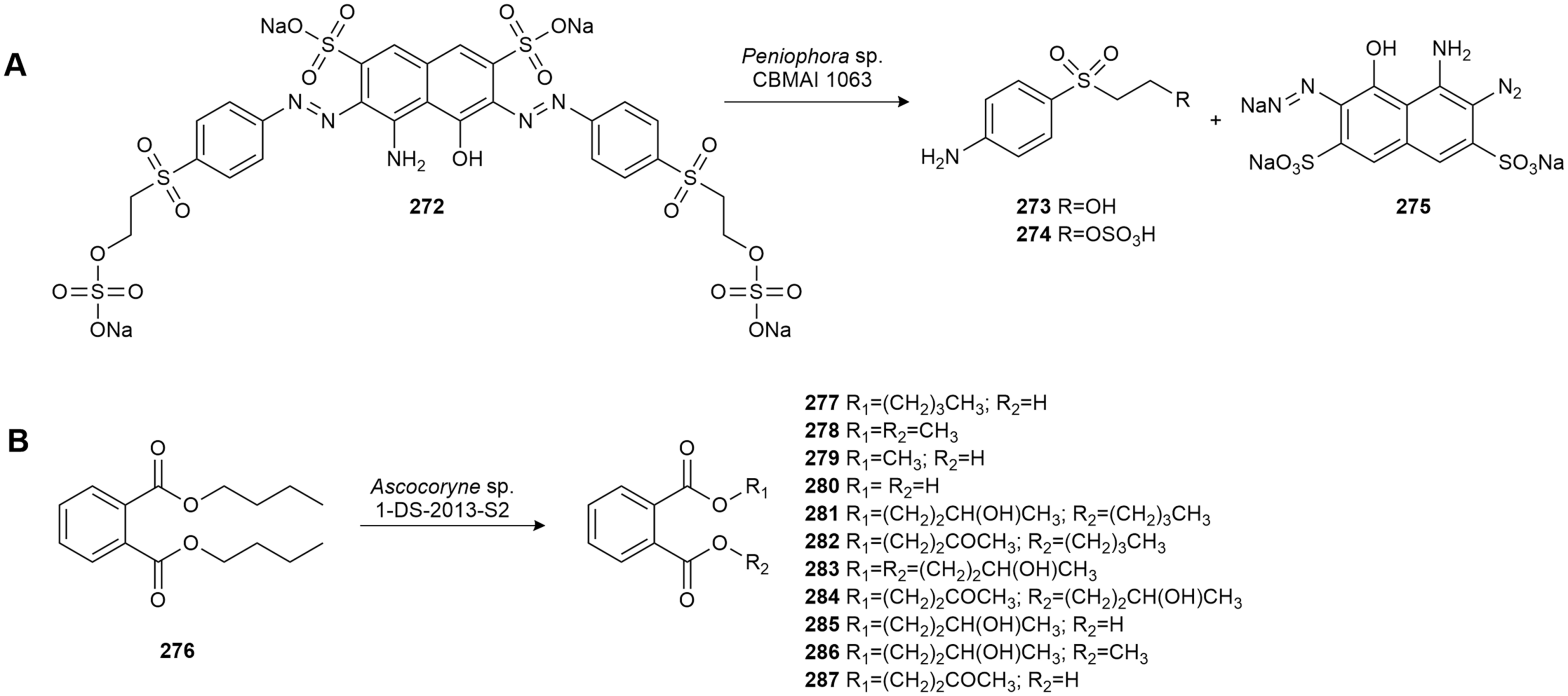
Biodegradation of a synthetic dye and a plastic polymer. **(A)** Reactive black 5 (**272**) dye biodegradation by *Peniophora* sp. CBMAI 1063 (Bonugli-Santos et al., 2016). **(B)** Biodegradation of di-*n*-butyl phthalate (**276**) plastic polymer by *Ascocoryne* sp. 1-DS-2013-S2 (Carstens et al., 2020).

One of the most extensively used dyes in the textile industry is reactive black 5 (RB5, **272**), which belongs to the group of reactive azo dyes. It decolorization in saline conditions by *Peniophora* sp. CBMAI 1063, a marine-derived fungus isolated from the Brazilian sponge *A. viridis*, was highly efficient (98% after 7 days). Most of the dye (80%) was consumed early in the process (24 h), when increased manganese peroxidase (MnP) gene expression and significant enzyme activity was observed in *Peniophora* sp. CBMAI 1063. RB5 was biodegraded to compounds **273**–**275**, formed by cleavage of both azo bridges (Figure 13A). Results from *Salmonella*/microsome assay (AMES) with the strains TA98 and TA100 test revealed that the biodegradation products did not exhibited mutagenic risk (Bonugli-Santos et al., 2016).

Remazol brilliant blue R (RBBR), also known as reactive blue 19, is another important dye used in the textile industry. It is a derivative of anthraquinone and belongs to an important class of toxic organo-contaminants. Its decolorization by marine-derived fungi has been extensively studied. da Silva et al. (2008) reported an efficient decolorization of RBBR after 12 days by the filamentous fungi *P. citrinum* CBMAI 853 (100%), *Aspergillus sulphureus* CBMAI 849 (95%), *C. cladosporioides* CBMAI 857 (93%) and *Trichoderma* sp. CBMAI 852 (89%), isolated from Brazilian zoanthid. Similarly, the basidiomycete fungi *Tinctoporellus* sp. CMBAI 1061, *Marasmiellus* sp. CBMAI 1062 and *Peniophora* sp. CBMAI 1063, isolated from the Brazilian sponges *A. viridis* and *D. reticulatum*, decolorized up to 100% of the dye, strain CBMAI 1061 being the most efficient. The three marine-derived basidiomycetes showed high manganese peroxidase and lignin peroxidase activity in non-saline and saline conditions. The dye was also completely removed by the crude enzymatic extracts of CBMAI 1061 and CBMAI 1063 after 4 and 24 h, respectively thus confirming the enzymatic biodegradation of RBBR by both fungi, while adsorption by the mycelium must be considered for strain CBMAI 1062 (Bonugli-Santos et al., 2012).

The basidiomycete fungus *F. flavus* (Klotzsch) Ryvarden (strain 312), isolated from decaying seagrass blades from the western coast of India, effectively degraded Congo red, Poly-B, Poly-R, RBBR, and azure B in a low nitrogen medium with 50% artificial water, while decolorization of brilliant green was relatively less efficient. On this medium, *F. flavus* produced three major classes of extracellular lignin-modifying enzymes: manganese-dependent peroxidase, lignin peroxidase, and laccase. For Poly-R and RBBR dyes, a direct correlation was observed between the percentage of decolorization and MnP activity, suggesting this enzyme’s involvement in this process (Raghukumar et al., 1999; Raghukumar, 2000). Similarly, a strain of *F. flavus* isolated from decaying *Thallasodendon ciliatum* seagrass collected in the Western Indian Ocean off Tanzania, was able to almost completely decolorize the synthetic dyes RBBR, Congo red, Brilliant green, Reactive black, and Reactive yellow in a low-carbon culture medium. The fungal filtrate exhibited maximum lignin peroxidase, manganese peroxidase and laccase activities. The lignin peroxidase of *F. flavus* was purified by ion exchange chromatography. This study confirmed extracellular enzymes from *F. flavus* to be potential degraders of organic pollutants (Mtui and Nakamura, 2008).

### 6.4. Plastic polymers

Plastics are synthetic organic polymers of high molecular mass that are widely produced around the world. Their global production has tripled in the last 25 years. Moreover, most of the plastics produced are single-use products that end up discarded in our natural environments causing serious pollution problems. This has negatively affected life on earth by leaching into the soil and increasing greenhouse emissions. The damage caused by plastic waste in the aquatic environment is also of great concern due to its major impact on marine biota (Amobonye et al., 2021).

Multiple efforts have been made to identify and isolate microorganisms capable of breaking down synthetic polymers. One such example is the marine fungus *Zalerion maritimum* ATTC 34329 from Portuguese coastal waters which managed to biodegrade polyethylene (PE) pellets in a minimum growth medium resulting in a decrease in pellet mass and size. FTIR-ATR and NMR spectra of freeze-dried samples of *Z. maritimum* showed a decrease in lipid and protein concentration and an increase in carbohydrate content correlating with time of exposure to microplastics, suggesting that the fungus uses them as a substrate (Paço et al., 2017).

Similarly, shaking cultures of the fungi *Aspergillus glaucus* and *A. niger*, isolated from mangrove soil off the southeast coast of India, degraded 28.80 and 17.35% of polyethylene bags, respectively, after 1 month. These results suggest that mangrove soil is a good source of microorganisms capable of degrading polyethylene (Kathiresan, 2003).

Among the different grades of polyethylene, the most important are low density polyethylene (LDPE), high density polyethylene (HDPE) and linear low density polyethylene (LLDPE). They differ in the number and size of the branches. HDPE has minimal branching of its polymer chains, while LDPE and LLDPE have more branching. In the case of LLDPE the branches are shorter.^1^ Among these grades, HDPE is the most commonly found non-degradable solid waste, thus posing a severe environmental threat. Its biodegradation by the fungi *Aspergillus tubingensis* VRKPT1 and *A. flavus* VRKPT2, isolated from the polyethylene waste dumped in the coastal area of the Gulf of Mannar in India, has been studied. Based on HDPE film weight loss and FT-IR spectrophotometric analysis, both strains were capable of degrading HDPE without any pre-treatment or pro-oxidant additives. Among these two strains, colonization, biofilm formation and biodegradation of HDPE film by *A. flavus* VRKPT2 was higher than by *A. tubingensis* VRKPT1. Also, during the experiment the smooth surface of the HDPE film became rough and brittle suggesting enzyme activity (Devi et al., 2015).

Pramila (2011) examined the ability of the fungi *A. versicolor* SB and *Aspergillus* sp. SD, isolated from seawater collected from the Bay of Bengal in India, to degrade LDPE strips. Based on the results of the Sturn test which measures the amount of CO_2_ released during the growth period, the isolate SB degraded 77% of the LDPE strips while the isolate SD degraded 83%, thus proving the efficiency of both as biodegradation agents. The LDPE film colonized by the fungi underwent structural changes such as the formation of pits, cracks and tiny holes, providing strong evidence of biodegradation.

Thermoplastic polyhydroxyalkanoates (PHAs) are produced by bacteria from renewable resources and could replace conventional plastics because they are biodegradable. The most common PHAs are poly-*β*-hydroxybutyrate (PHB), commercially available as BIOPOL®, and its copolymer with poly-*β*-hydroxyvalerate (PHV), known as PHB-co-PHV (Gonda et al., 2000; Matavulj and Molitoris, 2009). Gonda et al. (2000) investigated the degradation of BIOPOL^®^ by two deep sea isolates, the filamentous fungus *Aspergillus ustus* and the yeast *Rhodosporidium sphaerocarpum*, and two marine surface yeasts, *Candida guillermondii* and *Debaryomyces hansenii*, under different hydrostatic pressures. Based on the clearing test of PHB-turbid agar medium and/or a spectrophotometric assay to determine PHB-depolymerase activity, they reported that all strains degraded PHB in solid medium under atmospheric conditions. In contrast, in liquid medium, the ability to remove the pollutant decreased with increasing pressure and disappeared beyond 30 MPa, demonstrating the poor self-cleaning capacity of deep marine habitats.

Plastics contain plasticisers and additives to improve certain properties, which also pose risks to the environment and human health. These include phthalate esters which are plastic additives widely used to provide flexibility in the manufacture of plastic products such as polyvinyl chloride, and as a common additive in a variety of consumer products. Carstens et al. (2020) studied the removal of di-*n*-butyl phthalate (**276**) and diethyl phthalate (DEP) by the Baltic Sea-derived fungus *Ascocoryne* sp. 1-DS-2013-S2, isolated from sand containing algal debris in the alluvial zone. This strain removed **276** to a greater extent than DEP, despite its lower aqueous solubility. Based on UPLC-QTOF-MS analysis, **276** was biotransformed into compounds **277–287** (Figure 13B; only one of the possible isomers detected for **281** and **283–286** is shown). The reactions included transesterification, hydrolytic de-esterification, and cytochrome P450-catalyzed monohydroxylation of DBP with subsequent further oxidation of derivatives.

### 6.5. Other persistent organic pollutants

**Figure 14:**
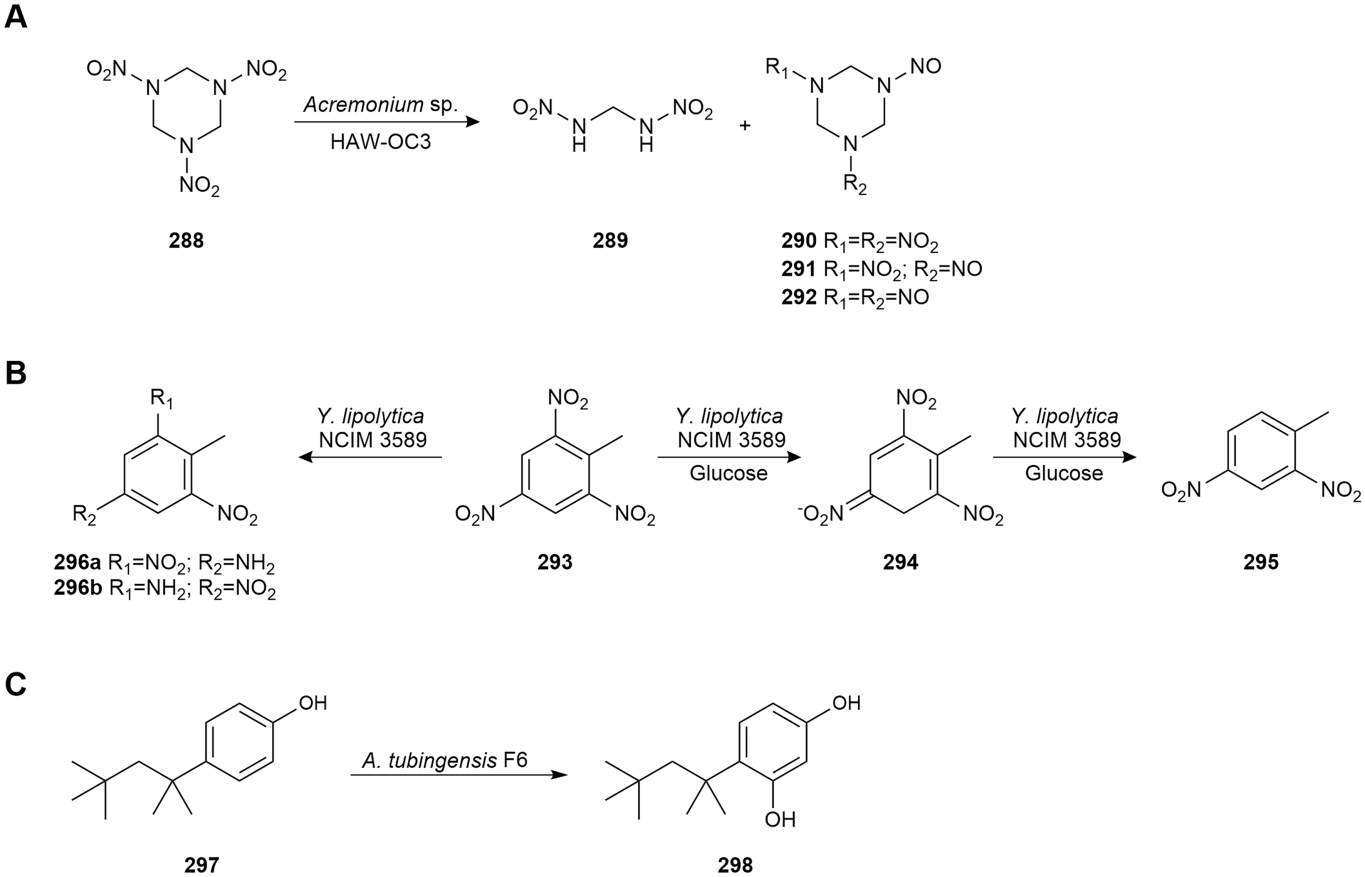
Biodegradations of other persistent organic pollutants. **(A)** Bioremediation of munitions compound hexahydro-1,3,5-trinitro-1,3,5-triazine (**288**) by *Acremonium* sp. HAW-OC3 (Bhatt et al., 2006). **(B)** Biodegradation of the explosive TNT (**293**) by *Yarrowia lipolytica* NCIM 3589 (Jain et al., 2004). **(C)** Biodegradation of the chemical 4-*tert*-octylphenol (**297**) by *Aspergillus tubingensis* F6 (Kuzikova et al., 2020).

Various naval military activities have resulted in the accumulation of unexploded ordnances in the marine environment which constitutes a major source of pollution due to their presence in sediments and aquatic organisms. Hexahydro-1,3,5-trinitro-1,3,5-triazine (**288**) is a typical munitions compound widely used by many naval defense departments around the world. Bhatt et al. (2006) reported its biodegradation by the marine fungal strains *Rhodotorula* sp. HAW-OCF1 (40% rate), *Bullera* sp. HAW-OCF2 (35%), *Acremonium* sp. HAW-OC3 (75%), and *Penicillium* sp. HAW-OCF5 (45%), isolated from sediments collected from Hawaiian coastal region. The biotransformation of **288** by strain HAW-OC3 produced methylenedinitramine (**289**), hexahydro-1-nitroso-3,5-dinitro-1,3,5-triazine (**290**), and traces of hexahydro-1,3-dinitroso-5-nitro-1,3,5-triazine (**291**) and hexahydro-1,3,5-trinitroso-1,3,5-triazine (**292**), in addition to N_2_O, CO_2_, or HCHO. These results suggest that the pollutant was biodegraded both by direct ring cleavage to form **289** and by reduction to **290** prior to ring cleavage (Figure 14A).

Another highly toxic explosive used in military shells, bombs and grenades is 2,4,6-trinitrotoluene (TNT, **293**). This pollutant was completely biodegraded by the tropical marine yeast *Y. lipolytica* NCIM 3589 in a YNB medium (Yeast Nitrogen Base containing amino acids and ammonium sulfate) with glucose after 48 h. Under these conditions, the yeast was able to reduce the aromatic ring of TNT to form the hydride-Meisenheimer complex **294**, which was subsequently denitrated to 2,4-dinitrotolueno (**295**), a compound that, in turn, could easily be degraded by monooxygenases of other microbes. In the absence of glucose, the yeast preferentially reduced the nitro groups of TNT to produce the aminodinitrotoluene isomers (**296**; Figure 14B; Jain et al., 2004).

Antibiotics are widely used to promote growth in intensive aquaculture. Oxytetracycline is one of the most frequently used antibiotics in the salmon industry to control bacterial infections due to its low cost and high efficacy. Excessive use of this antibiotic has serious detrimental effects on the environment which is why its bioremediation by marine fungi isolated from sediments collected in the vicinity of salmon farming areas has been evaluated. The isolates *Penicillium commune*, *Epicoccum nigrum*, *T. harzianum*, *A. terreus*, and *Beauveria bassiana* were able to degrade between 68.2 and 78.3% of oxytetracycline in liquid medium after 15 days (Ahumada-Rudolph et al., 2016).

Olsalazine is a highly lethal and toxic drug at concentrations above permitted levels in humans and animals. Its indiscriminate disposal into the environment poses a threat to human health and natural ecosystems. Efforts to completely eliminate the drug in an eco-friendly manner have proven to be quite arduous due to the recalcitrant nature of the azo bond in its structure. Bankole et al. (2021a) reported the degradation of 89.43% of olsalazine in 7 days by the marine-derived fungus *Aspergillus aculeatus* MT492456, isolated from saline soil samples collected off the Atlantic Ocean coast of Lagos. Biodegradation was carried out in the presence of the redox mediators azino-bis(3-ethylbenzothiazoline-6-sulphonic acid), *p*-coumaric acid and 1-hydroxybenzotriazole, which promoted electron transfer during osalazine oxidation.

Another environmental pollutant of great concern due to its potential health risks is formaldehyde which is widely used in the chemical industry as a disinfectant and in marine aquaculture. This chemical was completely degraded after 7 days by the fungus *P. chrysogenum* DY-F2, isolated from deep-sea sediments in the eastern Pacific. During biodegradation, formic acid was detected as an intermediate, indicating that formaldehyde was metabolized by the sequential action of formaldehyde dehydrogenase and then formic acid dehydrogenase (Luo et al., 2014).

Long-chain alkylphenols are also priority environmental contaminants due to their widespread application and their toxicity to the hormonal system of many organisms, including humans. These chemicals have especially been detected in aquatic environments where they tend to accumulate in seabed sediments. It was recently discovered that the marine fungus *A. tubingensis* F6, isolated from the seabed of the Gulf of Finland, is resistant to the toxicity of alkylphenols so its ability to remove this contaminant was evaluated. This strain was able to degrade 91 and 96% of 4-*tert*-octylphenol (**297**) and nonylphenol, respectively, after 120 h. According to GC–MS analysis, the main intermediate product in the biodegradation of 4-*tert*-octylphenol (**297**) was *p*-*tert*-octylresorcinol (**298**; Figure 14C). The filtrates of the fungal cultures after biodegradation of both alkylphenols by *A. tubingensis* F6 did not show toxicity in the assay with *Paramecium caudatum* Ehrenberg as a bioindicator, confirming the potential biotechnological application of this fungus in wastewater bioremediation (Kuzikova et al., 2020).

Polychlorinated biphenyls are persistent organic pollutants exhibiting high chemical stability and a tendency to persist in the environment and the human body. Although they were banned decades ago, they still pose a serious threat to human health and wildlife. Nikolaivits et al. (2021) evaluated the bioremediation of 2,4,5-trichlorobiphenyl by 104 fungal strains isolated from marine invertebrates. *Aspergillus* sp. TM122-S2, *Penicillium* sp. TM125-S3, *Cladosporium* sp. TM138-S3, *Purpureocillium lilacinum* TM138-S4, *Alternaria* sp. TM141-S1, *Aspergillus* sp. ML136-S2, *Aspergillus fumigatus* ML138-S2, and *P. steckii* MLm53-S3 exhibited bioconversion rates of the pollutant above 98.5%. Of these, *Cladosporium* sp. TM138-S3 exhibited the highest laccase activity, i.e., the enzyme most frequently involved in the removal of aromatic contaminants. Two enzymes exhibiting laccase activity, Lac1 y Lac2, were isolated from the culture broth of this strain through ion-exchange chromatography. Lac2 exhibited an improved ability to remove 2,4,5-trichlorobiphenyl (up to 71.2%) in the presence of mediators.

Organobromines are another persistent organic pollutant widely used in industry. These compounds are toxic, recalcitrant and tend to bioaccumulate in the environment. The marine ecosystem is known to contain significant amounts of organobromines, which may have led to the adaptation of marine microorganisms to these chemicals, making them promising candidates for bioremediation. For example, the tropical marine yeast *Y. lipolytica* NCIM 3589 is able to aerobically degrade bromoalkanes of differing carbon chain lengths and bromide group degrees and positions. Specifically, this yeast was able to degrade 27.3, 21.9, 18.0, and 38.3% of 2-bromopropane, 1-bromobutane, 1,5-dibromopentane, and 1-bromodecane, respectively. GC–MS analyses showed that the bromoalkanes were hydrolytically dehalogenated to give the corresponding alcohols, which were subsequently metabolized to fatty acids and finally into CO_2_. In this study, an inducible extracellular dehalogenase responsible for removal of bromide was detected (Vatsal et al., 2015).

## 7. Conclusion

Biotransformation has proven to be a valuable tool in the production of fine chemicals, in particular of enantiomerically pure compounds. Their application in chemical synthesis thus presents a golden opportunity for the development of chemical and pharmaceutical industrial processes.

The search for new biocatalysts has been growing significantly in recent years in order to obtain novel derivatives with enhanced properties for new drugs, agrochemicals and fragrances which are difficult to obtain using conventional synthetic methods. Thus, the screening of new fungal strains with interesting enzymatic activities has become absolutely necessary.

Marine fungi have been used in biotransformation processes of a large number of different compounds such as terpenes, steroids, polyketides, etc. producing various modifications in the substrate. Thus, these microorganisms were employed as biocatalysts for different reactions, including reduction, oxidation, hydroxylation, hydrolysis, dehalogenation, elimination, cyclization, rearrangement, etc.

Specifically, bioactive natural products have been widely used as substrates by these fungi to modify their structures with a view to enhancing their activity. Thus, the biotransformation of these compounds has proved to be a useful tool in their chemistry, especially stereoselective hydroxylation at deactivated positions. A very interesting and rare reaction is fungal mannosidation which was produced in terpenes by marine fungi for the first time.

Non-natural products are often biotransformed for use as intermediates in the synthesis of pharmaceutical or agrochemical compounds. For example, different marine fungi have been used to produce stereoselective carbonyl or double bond reductions to obtain enantioenriched compounds as the main biotransformation reaction involved. Moreover, organonitriles are usually hydrolysed by marine fungi to the corresponding enantiopure carboxylic acids. This is very interesting in terms of the use of these fungi in biodegradation processes.

Marine fungi adapt to extreme environments and have shown considerable potential for the detoxification of toxic and recalcitrant compounds present in wastewater, soils, sediments and solid waste. They have an arsenal of enzymes that can be harnessed to transform pollutants into less environmentally toxic compounds.

Although there are already numerous investigations focused on these microorganisms, the screening of new fungal strains with interesting enzymatic activities is necessary for the degradation of new pollutants resulting from increasing levels of industrial pollution. Furthermore, as most research on fungal bioremediation has been conducted under laboratory conditions, it is necessary to scale the process to contaminated environments where all natural variables are considered.

The main limiting factor for the biodegradation of organopollutants is their solubility rate. Therefore, the use of biosurfactants represents an interesting tool for their bioremediation. Since biosurfactants can be synthesized by many microorganisms (terrestrial and marine) they can be employed in integrated bioremediation protocols.

The heterogeneous and complex composition of marine sediments makes total remediation of pollutants by a single method very difficult. Therefore, integrated approaches, i.e., the combined use of traditional and bioremediation methods, or stand-alone bioremediation using a selected consortium of fungi, bacteria and biosurfactants acting synergistically, are more promising solutions.

## Author contributions

JA and RD-P conceived and coordinated the drafting of the manuscript. All authors conducted literature review, formulated and wrote the manuscript, contributed to the article, and approved the submitted version.

## Funding

This work has been co-financed by the European Union under the 2014–2020 ERDF Operational Program and by the Department of Economic Transformation, Industry, Knowledge, and Universities of the Regional Government of Andalusia. Project reference FEDER-UCA18-105749.

## Conflict of interest

The authors declare that the research was conducted in the absence of any commercial or financial relationships that could be construed as a potential conflict of interest.

## Publisher’s note

All claims expressed in this article are solely those of the authors and do not necessarily represent those of their affiliated organizations, or those of the publisher, the editors and the reviewers. Any product that may be evaluated in this article, or claim that may be made by its manufacturer, is not guaranteed or endorsed by the publisher.

## Footnotes

^1^https://www.usplastic.com/knowledgebase/article.aspx?contentkey=508. Revised 15/11/2022.

## References

[ref1] Ahumada-Rudolph R. Novoa V. Sáez K. Martínez M. Rudolph A. Torres-Diaz C.. (2016). Marine fungi isolated from Chilean fjord sediments can degrade oxytetracycline. Environ. Monit. Assess. 188:468. doi: 10.1007/s10661-016-5475-0, PMID: 27418075

[ref2] Alvarenga N. Birolli W. G. Nitschke M. De O. Rezende M. O. Seleghim M. H. R.. (2015). Biodegradation of chlorpyrifos by whole cells of marine-derived fungi *aspergillus sydowii* and *Trichoderma* sp. J. Microb. Biochem. Technol. 07, 133–139. doi: 10.4172/1948-5948.1000194

[ref3] Alvarenga N. Birolli W. G. Seleghim M. H. R. Porto A. L. M. (2014). Biodegradation of methyl parathion by whole cells of marine-derived fungi *aspergillus sydowii* and *Penicillium decaturense*. Chemosphere 117, 47–52. doi: 10.1016/j.chemosphere.2014.05.069, PMID: 24955826

[ref4] Alvarenga N. Porto A. L. M. (2017). Stereoselective reduction of 2-azido-1-phenylethanone derivatives by whole cells of marine-derived fungi applied to synthesis of enantioenriched *β*-hydroxy-1,2,3-triazoles. Biocatal. Biotransformation 35, 388–396. doi: 10.1080/10242422.2017.1352585

[ref5] Álvarez-Barragán J. Cravo-Laureau C. Wick L. Y. Duran R. (2021). Fungi in PAH-contaminated marine sediments: cultivable diversity and tolerance capacity towards PAH. Mar. Pollut. Bull. 164:112082. doi: 10.1016/j.marpolbul.2021.112082, PMID: 33524832

[ref6] Amobonye A. Bhagwat P. Singh S. Pillai S. (2021). Plastic biodegradation: frontline microbes and their enzymes. Sci. Total Environ. 759:143536. doi: 10.1016/j.scitotenv.2020.143536, PMID: 33190901

[ref7] Arun A. Raja P. P. Arthi R. Ananthi M. Kumar K. S. Eyini M. (2008). Polycyclic aromatic hydrocarbons (PAHs) biodegradation by basidiomycetes fungi, *pseudomonas* isolate, and their cocultures: comparative in vivo and in silico approach. Appl. Biochem. Biotechnol. 151, 132–142. doi: 10.1007/s12010-008-8160-0, PMID: 18975143

[ref8] Bankole P. O. Semple K. T. Jeon B.-H. Govindwar S. P. (2021a). Impact of redox-mediators in the degradation of olsalazine by marine-derived fungus, *aspergillus aculeatus* strain bpo2: response surface methodology, laccase stability and kinetics. Ecotoxicol. Environ. Saf. 208:111742. doi: 10.1016/j.ecoenv.2020.111742, PMID: 33396068

[ref9] Bankole P. O. Semple K. T. Jeon B. H. Govindwar S. P. (2021b). Biodegradation of fluorene by the newly isolated marine-derived fungus, *Mucor irregularis* strain bpo1 using response surface methodology. Ecotoxicol. Environ. Saf. 208:111619. doi: 10.1016/j.ecoenv.2020.111619, PMID: 33396139

[ref10] Bhatt J. K. Ghevariya C. M. Dudhagara D. R. Rajpara R. K. Dave B. P. (2014). Application of response surface methodology for rapid chrysene biodegradation by newly isolated marine-derived fungus *Cochliobolus lunatus* strain CHR4D. J. Microbiol. 52, 908–917. doi: 10.1007/s12275-014-4137-6, PMID: 25359268

[ref11] Bhatt M. Zhao J.-S. Halasz A. Hawari J. (2006). Biodegradation of hexahydro-1,3,5-trinitro-1,3,5-triazine by novel fungi isolated from unexploded ordnance contaminated marine sediment. J. Ind. Microbiol. Biotechnol. 33, 850–858. doi: 10.1007/s10295-006-0136-x, PMID: 16703352

[ref12] Bhatti H. N. Khera R. A. (2012). Biological transformations of steroidal compounds: A review. Steroids 77, 1267–1290. doi: 10.1016/j.steroids.2012.07.018 22910289

[ref13] Birolli W. G. Alvarenga N. Seleghim M. H. R. Porto A. L. M. (2016). Biodegradation of the pyrethroid pesticide esfenvalerate by marine-derived fungi. Mar. Biotechnol. 18, 511–520. doi: 10.1007/s10126-016-9710-z, PMID: 27381569

[ref14] Birolli W. G. Alvarenga N. Vacondio B. Seleghim M. H. R. Porto A. L. M. (2014). Growth assessment of marine-derived fungi in the presence of esfenvalerate and its main metabolites. J. Microb. Biochem. Technol. 06, 260–267. doi: 10.4172/1948-5948.1000154

[ref15] Birolli W. G. de Santos D. Alvarenga N. Garcia A. C. F. S. Romão L. P. C. Porto A. L. M. (2018a). Biodegradation of anthracene and several PAHs by the marine-derived fungus *Cladosporium* sp. CBMAI 1237. Mar. Pollut. Bull. 129, 525–533. doi: 10.1016/j.marpolbul.2017.10.023, PMID: 29055563

[ref001] Birolli W. G. Lima R. N. Porto A. L. M. (2019). Applications of marine-derived microorganisms and their enzymes in biocatalysis and biotransformation, the underexplored potentials. Front. Microbiol. 10, 1453. doi: 10.3389/fmicb.2019.01453 31481935 PMC6710449

[ref16] Birolli W. G. Ferrreira I. M. Jimenez D. E. Q. Silva B. N. M. Silva B. V. Pinto A. C.. (2017). First asymmetric reduction of isatin by marine-derived fungi. J. Braz. Chem. Soc. 28, 1023–1029. doi: 10.21577/0103-5053.20160256

[ref17] Birolli W. G. Vacondio B. Alvarenga N. Seleghim M. H. R. Porto A. L. M. (2018b). Enantioselective biodegradation of the pyrethroid (±)-lambda-cyhalothrin by marine-derived fungi. Chemosphere 197, 651–660. doi: 10.1016/j.chemosphere.2018.01.054, PMID: 29407829

[ref18] Birolli W. G. Yamamoto K. Y. de Oliveira J. R. Nitschke M. Seleghim M. H. R. Porto A. L. M. (2015). Biotransformation of dieldrin by the marine fungus *Penicillium miczynskii* CBMAI 930. Biocatal. Agric. Biotechnol. 4, 39–43. doi: 10.1016/j.bcab.2014.06.002

[ref19] Birolli W. G. Zanin L. L. Jimenez D. E. Q. Porto A. L. M. (2020). Synthesis of Knoevenagel adducts under microwave irradiation and biocatalytic ene-reduction by the marine-derived fungus *Cladosporium* sp. CBMAI 1237 for the production of 2-cyano-3-phenylpropanamide derivatives. Mar. Biotechnol. 22, 317–330. doi: 10.1007/s10126-020-09953-8, PMID: 32124098

[ref20] Bjerregaard-Andersen K. Sommer T. Jensen K. Jochimsen B. Etzerodt M. Morth P. (2014). A proton wire and water channel revealed in the crystal structure of isatin hydrolase. J. Biol. Chem. 289, 21351–21359. doi: 10.1074/jbc.M114.568824, PMID: 24917679 PMC4118100

[ref21] Bonugli-Santos R. C. dos Santos Vasconcelos M. R. Passarini M. R. Z. Vieira G. A. L. Lopes V. C. P. Mainardi P. H.. (2015). Marine-derived fungi: diversity of enzymes and biotechnological applications. Front. Microbiol. 6:269. doi: 10.3389/fmicb.2015.00269, PMID: 25914680 PMC4392690

[ref22] Bonugli-Santos R. C. Durrant L. R. Sette L. D. (2012). The production of ligninolytic enzymes by marine-derived basidiomycetes and their biotechnological potential in the biodegradation of recalcitrant pollutants and the treatment of textile effluents. Water Air Soil Pollut. 223, 2333–2345. doi: 10.1007/s11270-011-1027-y

[ref23] Bonugli-Santos R. C. Vieira G. A. L. Collins C. Fernandes T. C. C. Marin-Morales M. A. Murray P.. (2016). Enhanced textile dye decolorization by marine-derived basidiomycete *Peniophora* sp. CBMAI 1063 using integrated statistical design. Environ. Sci. Pollut. Res. 23, 8659–8668. doi: 10.1007/s11356-016-6053-2, PMID: 26797957

[ref24] Campos-Góngora E. Palande A. S. León-Ramirez C. Pathan E. K. Ruiz-Herrera J. Deshpande M. V. (2018). Determination of the effect of polyamines on an oil-degrading strain of *Yarrowia lipolytica* using an odc minus mutant. FEMS Yeast Res. 18:73. doi: 10.1093/femsyr/foy073, PMID: 29982373

[ref25] Capotorti G. Cesti P. Lombardi A. (2005). Formation of sulfate conjugates metabolites in the degradation of phenanthrene, anthracene, pyrene and benzo[a]pyrene by the ascomycete *aspergillus terreus*. Polycycl Aromat Comp 25, 197–213. doi: 10.1080/10406630590950273

[ref26] Carstens L. Cowan A. R. Seiwert B. Schlosser D. (2020). Biotransformation of phthalate plasticizers and bisphenol A by marine-derived, freshwater, and terrestrial fungi. Front. Microbiol. 11, 1–21. doi: 10.3389/fmicb.2020.00317, PMID: 32180766 PMC7059612

[ref27] Chen P.-N. Hao M.-J. Li H.-J. Xu J. Mahmud T. Lan W.-J. (2021). Biotransformations of anthranilic acid and phthalimide to potent antihyperlipidemic alkaloids by the marine-derived fungus *Scedosporium apiospermum* F41-1. Bioorg. Chem. 116:105375. doi: 10.1016/j.bioorg.2021.105375, PMID: 34563999

[ref28] Cheng X.-C. Varoglu M. Abrell L. Crews P. Lobkovsky E. Clardy J. (1994). Chloriolins A-C, chlorinated sesquiterpenes produced by fungal cultures separated from a jaspis marine sponge. J. Org. Chem. 59, 6344–6348. doi: 10.1021/jo00100a041

[ref29] D’Souza D. T. Tiwari R. Sah A. K. Raghukumar C. (2006). Enhanced production of laccase by a marine fungus during treatment of colored effluents and synthetic dyes. Enzym. Microb. Technol. 38, 504–511. doi: 10.1016/j.enzmictec.2005.07.005

[ref30] da Silva N. A. Birolli W. G. Seleghim M. H. R. Porto A. L. M. (2013). “Biodegradation of the organophosphate pesticide profenofos by marine fungi” in Applied bioremediation - active and passive approaches. eds. Patil Y. B. Rao P. (UK: IntechOpen), 150–180.

[ref31] da Silva M. Passarini M. R. Z. Bonugli R. C. Sette L. D. (2008). Cnidarian-derived filamentous fungi from Brazil: isolation, characterisation and RBBR decolourisation screening. Environ. Technol. 29, 1331–1339. doi: 10.1080/09593330802379466, PMID: 19149354

[ref32] de Jesus H. C. R. Jeller A. H. Debonsi H. M. Alves P. B. Porto A. L. M. (2017). Multiple monohydroxylation products from rac-camphor by marine fungus *Botryosphaeria* sp. isolated from marine alga *Bostrychia radicans*. J. Braz. Chem. Soc. 28, 498–504. doi: 10.21577/0103-5053.20160262

[ref33] de Matos I. L. Birolli W. G. Santos D. A. Nitschke M. Porto A. L. M. (2021a). Stereoselective reduction of flavanones by marine-derived fungi. Mol. Catal. 513:111734. doi: 10.1016/j.mcat.2021.111734

[ref34] de Matos I. L. Nitschke M. Porto A. L. M. (2019). Hydrogenation of halogenated 2′-hydroxychalcones by mycelia of marine-derived fungus *Penicillium raistrickii*. Mar. Biotechnol. 21, 430–439. doi: 10.1007/s10126-019-09893-y, PMID: 30895403

[ref35] de Matos I. L. Nitschke M. Porto A. L. M. (2021b). Regioselective and chemoselective biotransformation of 2′-hydroxychalcone derivatives by marine-derived fungi. Biocatal. Biotransformation, 41, 46–56. doi: 10.1080/10242422.2021.1956909

[ref36] de Oliveira J. R. Mizuno C. M. Seleghim M. H. R. Javaroti D. C. D. Rezende M. O. O. Landgraf M. D.. (2013). Biotransformation of phenylacetonitrile to 2-hydroxyphenylacetic acid by marine fungi. Mar. Biotechnol. 15, 97–103. doi: 10.1007/s10126-012-9464-1, PMID: 22790719

[ref37] de Oliveira J. R. Seleghim M. H. R. Porto A. L. M. (2014). Biotransformation of methylphenylacetonitriles by Brazilian marine fungal strain *aspergillus sydowii* CBMAI 934: eco-friendly reactions. Mar. Biotechnol. 16, 156–160. doi: 10.1007/s10126-013-9534-z, PMID: 24057165

[ref38] de Paula S. F. C. Porto A. L. M. (2020). Cascate reactions of progesterone by mycelia and culture broth from marine-derived fungus *aspergillus sydowii* CBMAI 935. Biocatal. Agric. Biotechnol. 25:101546. doi: 10.1016/j.bcab.2020.101546

[ref39] de Paula S. F. C. Rosset I. G. Porto A. L. M. (2021). Hydroxylated steroids in C-7 and C-15 positions from progesterone bio-oxidation by the marine-derived fungus *Penicillium oxalicum* CBMAI 1996. Biocatal. Agric. Biotechnol. 37:102167. doi: 10.1016/j.bcab.2021.102167

[ref40] de Queiroz T. M. Ellena J. Porto A. L. M. (2020). Biotransformation of ethinylestradiol by whole cells of Brazilian marine-derived fungus *Penicillium oxalicum* CBMAI 1996. Mar. Biotechnol. 22, 673–682. doi: 10.1007/s10126-020-09989-w, PMID: 32833111

[ref41] de Souza J. M. Santos M. F. C. Pedroso R. C. N. Pimenta L. P. Siqueira K. A. Soares M. A.. (2021). Optimization of (−)-cubebin biotransformation to (−)-hinokinin by the marine fungus *Absidia coerulea* 3A9. Arch. Microbiol. 203, 4313–4318. doi: 10.1007/s00203-021-02417-0, PMID: 34110481

[ref42] Dell’ Anno F. Rastelli E. Sansone C. Brunet C. Ianora A. Dell’ Anno A. (2021). Bacteria, fungi and microalgae for the bioremediation of marine sediments contaminated by petroleum hydrocarbons in the omics era. Microorganisms 9:1695. doi: 10.3390/MICROORGANISMS9081695 34442774 PMC8400010

[ref43] Devi R. S. Kannan V. R. Nivas D. Kannan K. Chandru S. Antony A. R. (2015). Biodegradation of HDPE by *aspergillus* spp. from marine ecosystem of gulf of Mannar, India. Mar. Pollut. Bull. 96, 32–40. doi: 10.1016/j.marpolbul.2015.05.050, PMID: 26006776

[ref44] Dong X. Jiang W. Li C. Ma N. Xu Y. Meng X. (2015). Patulin biodegradation by marine yeast *Kodameae ohmeri*. *Food Addit. Contam*. Part A 32, 352–360. doi: 10.1080/19440049.2015.1007090, PMID: 25585640

[ref45] dos Santos V. M. R. Donnici C. L. DaCosta J. B. N. Caixeiro J. M. R. (2007). Compostos organofosforados pentavalentes: histórico, métodos sintéticos de preparação e aplicações como inseticidas e agentes antitumorais. Quim Nova 30, 159–170. doi: 10.1590/S0100-40422007000100028

[ref46] Edwards F. Tchounwou P. (2005). Environmental toxicology and health effects associated with methyl parathion exposure – A scientific review. Int. J. Environ. Res. Public Health 2, 430–441. doi: 10.3390/ijerph2005030007 16819098

[ref47] Ferreira I. M. de Vasconcellos S. P. da Cruz J. B. Comasseto J. V. Porto A. L. M. Rocha L. C. (2015a). Hydrogenation of bis-*α*,*β*-unsaturated enones mediated by filamentous fungi. Biocatal. Agric. Biotechnol. 4, 144–149. doi: 10.1016/j.bcab.2015.03.001

[ref48] Ferreira I. M. Fiamingo A. Campana-Filho S. P. Porto A. L. M. (2020). Biotransformation of (*E*)-2-methyl-3-phenylacrylaldehyde using mycelia of *Penicillium citrinum* CBMAI 1186, both free and immobilized on chitosan. Mar. Biotechnol. 22, 348–356. doi: 10.1007/s10126-020-09954-7, PMID: 32080775

[ref49] Ferreira I. M. Meira E. B. Rosset I. G. Porto A. L. M. (2015b). Chemoselective biohydrogenation of *α*,*β*- and *α*,*β*,*γ*,*δ*-unsaturated ketones by the marine-derived fungus *Penicillium citrinum* CBMAI 1186 in a biphasic system. J. Mol. Catal. B Enzym. 115, 59–65. doi: 10.1016/j.molcatb.2015.01.017

[ref50] Ferreira I. M. Rocha L. C. Yoshioka S. A. Nitschke M. Jeller A. H. Pizzuti L.. (2014). Chemoselective reduction of chalcones by whole hyphae of marine fungus *Penicillium citrinum* CBMAI 1186, free and immobilized on biopolymers. Biocatal. Agric. Biotechnol. 3, 358–364. doi: 10.1016/j.bcab.2014.04.001

[ref51] Gonçalves M. F. M. Esteves A. C. Alves A. (2022). Marine fungi: opportunities and challenges. Encyclopedia 2, 559–577. doi: 10.3390/encyclopedia2010037

[ref52] Gonda K. E. Jendrossek D. Molitoris H. P. (2000). Fungal degradation of the thermoplastic polymer poly-*β*-hydroxybutyric acid (PHB) under simulated deep sea pressure. Hydrobiologia 426, 173–183. doi: 10.1023/A:1003971925285

[ref53] Gozari M. Alborz M. El-Seedi H. R. Jassbi A. R. (2021). Chemistry, biosynthesis and biological activity of terpenoids and meroterpenoids in bacteria and fungi isolated from different marine habitats. Eur. J. Med. Chem. 210:112957. doi: 10.1016/j.ejmech.2020.112957, PMID: 33160760

[ref54] Grossart H. Van den Wyngaert S. Kagami M. Wurzbacher C. Cunliffe M. Rojas-Jiménez K. (2019). Fungi in aquatic ecosystems. Nat. Rev. Microbiol. 17, 339–354. doi: 10.1038/s41579-019-0175-8 30872817

[ref55] Guerriero A. D’Ambrosio M. Pietra F. Cuomo V. Vanzanella F. (1988). Dendryphiellin A, the first fungal trinor-eremophilane. Isolation from the marine deuteromycete *Dendryphiella salina* (Sutherland). *Helv. Chim*. Acta 71, 57–61. doi: 10.1002/hlca.19880710107

[ref56] Haritash A. K. Kaushik C. P. (2009). Biodegradation aspects of polycyclic aromatic hydrocarbons (PAHs): A review. J. Hazard. Mater. 169, 1–15. doi: 10.1016/j.jhazmat.2009.03.137 19442441

[ref57] Jain M. R. Zinjarde S. S. Deobagkar D. D. Deobagkar D. N. (2004). 2,4,6-trinitrotoluene transformation by a tropical marine yeast, *Yarrowia lipolytica* NCIM 3589. Mar. Pollut. Bull. 49, 783–788. doi: 10.1016/j.marpolbul.2004.06.007, PMID: 15530522

[ref58] Jensen P. R. Fenical W. (1996). Marine bacterial diversity as a resource for novel microbial products. J. Ind. Microbiol. Biotechnol. 17, 346–351. doi: 10.1007/BF01574765

[ref59] Jimenez D. E. Q. Barreiro J. C. Dos Santos F. M. de Vasconcellos S. P. Porto A. L. M. Batista J. M. (2019). Enantioselective ene-reduction of *E*-2-cyano-3-(furan-2-yl) acrylamide by marine and terrestrial fungi and absolute configuration of (*R*)-2-cyano-3-(furan-2-yl) propanamide determined by calculations of electronic circular dichroism (ECD) spec. Chirality 31, 534–542. doi: 10.1002/chir.23078, PMID: 31197903

[ref60] Jimenez D. E. Q. Ferreira I. M. Birolli W. G. Fonseca L. P. Porto A. L. M. (2016). Synthesis and biocatalytic ene-reduction of Knoevenagel condensation compounds by the marine-derived fungus *Penicillium citrinum* CBMAI 1186. Tetrahedron 72, 7317–7322. doi: 10.1016/j.tet.2016.02.014

[ref61] Jin L. Quan C. Hou X. Fan S. (2016). Potential pharmacological resources: natural bioactive compounds from marine-derived fungi. Mar. Drugs 14:76. doi: 10.3390/md14040076, PMID: 27110799 PMC4849080

[ref62] Kathiresan K. (2003). Polythene and plastic-degrading microbes in an Indian mangrove soil. Rev. Biol. Trop. 51, 629–633. 15162769

[ref63] Koshimura M. Utsukihara T. Kawamoto M. Saito M. Horiuchi C. A. Kuniyoshi M. (2009). Biotransformation of bromosesquiterpenes by marine fungi. Phytochemistry 70, 2023–2026. doi: 10.1016/j.phytochem.2009.08.021, PMID: 19772936

[ref64] Kuban M. Öngen G. Bedir E. (2010). Biotransformation of cycloastragenol by *Cunninghamella blakesleeana* NRRL 1369 resulting in a novel framework. Org. Lett. 12, 4252–4255. doi: 10.1021/ol101642a, PMID: 20809612

[ref65] Kunteng W. Laiyou W. Wenjian Y. Danfeng Y. Houjin L. (2015). Hydroxylated biotransformation of flavone by marine fungi induced degradation of benzene and toluene. Chinese J. Appl. Chem. 32, 671–675. doi: 10.11944/j.issn.1000-0518.2015.06.140346

[ref66] Kuzikova I. Rybalchenko O. Kurashov E. Krylova Y. Safronova V. Medvedeva N. (2020). Defense responses of the marine-derived fungus *aspergillus tubingensis* to alkylphenols stress. Water Air Soil Pollut. 231:271. doi: 10.1007/s11270-020-04639-2

[ref67] Lalitha P. Reddy N. N. R. Arunalakshmi K. (2011). Decolorization of synthetic dyes by *aspergillus flavus*. Biorem. J. 15, 121–132. doi: 10.1080/10889868.2011.574651

[ref68] Lambert M. Kremer S. Anke H. (1995). Antimicrobial, phytotoxic, nematicidal, cytotoxic, and mutagenic activities of 1-hydroxypyrene, the initial metabolite in pyrene metabolism by the basidiomycete *Crinipellis stipitaria*. Bull. Environ. Contam. Toxicol. 55, 251–257. doi: 10.1007/BF00203017, PMID: 7579931

[ref69] Leutou A. S. Yang G. Nenkep V. N. Siwe X. N. Feng Z. Khong T. T.. (2009). Microbial transformation of a monoterpene, geraniol, by the marine-derived fungus *Hypocrea* sp. J. Microbiol. Biotechnol. 19, 1150–1152. doi: 10.4014/jmb.0904.04013, PMID: 19884773

[ref70] Li X. Kim Y. H. Jung J. H. Kang J. S. Kim D.-K. Choi H. D.. (2007). Microbial transformation of the bioactive sesquiterpene, cyclonerodiol, by the ascomycete *Penicillium* sp. and the actinomycete *Streptomyces* sp. *enzyme microb*. Technol. 40, 1188–1192. doi: 10.1016/j.enzmictec.2006.09.002

[ref71] Li H. Lan W. Lin Y. (2005). Biotransformation of 1,2,3,4-tetrahydronaphtalene by marine fungus *Hypoxylon oceanicum*. Fenxi Ceshi Xuebao 24, 45–47.

[ref72] Liu H. Chen B.-S. de Souza F. Z. R. Liu L. (2018a). A comparative study on asymmetric reduction of ketones using the growing and resting cells of marine-derived fungi. Mar. Drugs 16:62. doi: 10.3390/md16020062, PMID: 29443943 PMC5852490

[ref73] Liu H. de Souza F. Z. R. Liu L. Chen B.-S. (2018b). Immobilized and free cells of *Geotrichum candidum* for asymmetric reduction of ketones: stability and recyclability. Molecules 23:2144. doi: 10.3390/molecules23092144, PMID: 30150533 PMC6225435

[ref74] Liu H. de Souza F. Z. R. Liu L. Chen B.-S. (2018c). The use of marine-derived fungi for preparation of enantiomerically pure alcohols. Appl. Microbiol. Biotechnol. 102, 1317–1330. doi: 10.1007/s00253-017-8707-5, PMID: 29264774

[ref75] Liu H. Lei X.-L. Li N. Zong M.-H. (2013). Highly regioselective synthesis of betulone from betulin by growing cultures of marine fungus *Dothideomycete* sp. HQ 316564. J. Mol. Catal. B Enzym. 88, 32–35. doi: 10.1016/j.molcatb.2012.08.011

[ref76] Liu W.-C. Ren Y.-X. Hao A.-Y. Yu S. Shi X. Zhang X.-Q.. (2018d). The activities of wortmannilactones against helminth electron transport chain enzymes, structure-activity relationships, and the effect on *Trichinella spiralis* infected mice. J. Antibiot. (Tokyo) 71, 731–740. doi: 10.1038/s41429-018-0061-z, PMID: 29691485

[ref77] Luo J. J. Ding J. F. Li G. W. Zheng T. L. Luo Z. H. (2014). Characterization of a formaldehyde degrading fungus *Penicillium chrysogenum* DY-F2 isolated from deep sea sediment. Int. Biodeterior. Biodegrad. 89, 45–49. doi: 10.1016/j.ibiod.2013.12.019

[ref78] Mahajan M. Manek D. Vora N. Kothari R. K. Mootapally C. Nathani N. M. (2021). Fungi with high ability to crunch multiple polycyclic aromatic hydrocarbons (PAHs) from the pelagic sediments of gulfs of Gujarat. Mar. Pollut. Bull. 167:112293. doi: 10.1016/j.marpolbul.2021.112293, PMID: 33799152

[ref79] Martins M. P. Mouad A. M. Boschini L. Seleghim M. H. R. Sette L. D. Porto A. L. M. (2011). Marine fungi *aspergillus sydowii* and *Trichoderma* sp. catalyze the hydrolysis of benzyl glycidyl ether. Mar. Biotechnol. 13, 314–320. doi: 10.1007/s10126-010-9302-2, PMID: 20549284

[ref80] Martins M. P. Mouad A. M. Porto A. L. M. (2012). Hydrolysis of allylglycidyl ether by marine fungus *Trichoderma* sp. Gc1 and the enzymatic resolution of allylchlorohydrin by *Candida antarctica* lipase type B. Curr. Top. Catal. 10, 27–33.

[ref81] Martins M. P. Ouazzani J. Arcile G. Jeller A. H. de Lima J. P. F. Seleghim M. H. R.. (2015). Biohydroxylation of (−)-ambrox®, (−)-sclareol, and (+)-sclareolide by whole cells of Brazilian marine-derived fungi. Mar. Biotechnol. 17, 211–218. doi: 10.1007/s10126-015-9610-7, PMID: 25634054

[ref82] Matavulj M. Molitoris H. (2009). Marine fungi: degraders of poly-3-hydroxyalkanoate based plastic materials. Zb. Matice Srp. za Prir. Nauk, 116, 253–265. doi: 10.2298/ZMSPN0916253M

[ref83] Morais A. T. B. Ferreira I. M. Jimenez D. E. Q. Porto A. L. M. (2018). Synthesis of *α*-chloroacetophenones with NH_4_Cl/Oxone® in situ followed by bioreduction with whole cells of marine-derived fungi. Biocatal. Agric. Biotechnol. 16, 314–319. doi: 10.1016/j.bcab.2018.08.003

[ref84] Mouad A. M. de Oliveira A. L. L. Debonsi H. M. Porto A. L. M. (2015). Bioreduction of fluoroacetophenone derivatives by endophytic fungi isolated from the marine red alga *Bostrychia radicans*. Biocatalysis 1, 141–147. doi: 10.1515/boca-2015-0011

[ref85] Mouad A. M. Martins M. P. Romminger S. Debonsi H. M. Seleghim M. H. R. Oliveira A. L. L. de. (2012). Bioconversion of acetophenones by marine fungi isolated from marine algae *Bostrychia radicans* and *Sargassum* sp. Curr. Top. Biotechnol. 7, 13–19.

[ref86] Mtui G. Nakamura Y. (2008). Lignocellulosic enzymes from *Flavodon flavus*, a fungus isolated from Western Indian Ocean off the coast of Dar Es Salaam, Tanzania. African J. Biotechnol. 7, 3066–3072.

[ref87] Muñoz Solano D. Hoyos P. Hernáiz M. J. Alcántara A. R. Sánchez-Montero J. M. (2012). Industrial biotransformations in the synthesis of building blocks leading to enantiopure drugs. Bioresour. Technol. 115, 196–207. doi: 10.1016/j.biortech.2011.11.131, PMID: 22230779

[ref88] Namboori K. Pereira L. Merchant J. R. (1980). Fungal transformation of pregnenolone & progesterone with the marine fungus *Cladosporium herbarum*. Indian J. Biochem. Biophys. 17, 149–152. 7192690

[ref89] Nhi-Cong L. T. Mai C. T. N. Minh N. N. Ha H. P. Lien D. T. Tuan D. Van. (2016). Degradation of sec-hexylbenzene and its metabolites by a biofilm-forming yeast *Trichosporon asahii* B1 isolated from oil-contaminated sediments in Quangninh coastal zone, Vietnam. J. Environ. Sci. Health. 51, 267–275. doi: 10.1080/10934529.2015.1094351 26654204

[ref90] Nikolaivits E. Agrafiotis A. Baira E. Le Goff G. Tsafantakis N. Chavanich S. A.. (2020). Degradation mechanism of 2,4-dichlorophenol by fungi isolated from marine invertebrates. Int. J. Mol. Sci. 21:3317. doi: 10.3390/ijms21093317, PMID: 32392868 PMC7247547

[ref91] Nikolaivits E. Agrafiotis A. Termentzi A. Machera K. Le Goff G. Álvarez P.. (2019). Unraveling the detoxification mechanism of 2,4-dichlorophenol by marine-derived mesophotic symbiotic fungi isolated from marine invertebrates. Mar. Drugs 17:564. doi: 10.3390/md17100564, PMID: 31575010 PMC6835501

[ref92] Nikolaivits E. Dimarogona M. Fokialakis N. Topakas E. (2017). Marine-derived biocatalysts: importance, accessing, and application in aromatic pollutant bioremediation. Front. Microbiol. 8:265. doi: 10.3389/fmicb.2017.00265, PMID: 28265269 PMC5316534

[ref93] Nikolaivits E. Siaperas R. Agrafiotis A. Ouazzani J. Magoulas A. Gioti Α.. (2021). Functional and transcriptomic investigation of laccase activity in the presence of PCB29 identifies two novel enzymes and the multicopper oxidase repertoire of a marine-derived fungus. Sci. Total Environ. 775:145818. doi: 10.1016/j.scitotenv.2021.145818, PMID: 33631558

[ref94] Ortega S. N. Nitschke M. Mouad A. M. Landgraf M. D. Rezende M. O. O. Seleghim M. H. R.. (2011). Isolation of Brazilian marine fungi capable of growing on DDD pesticide. Biodegradation 22, 43–50. doi: 10.1007/s10532-010-9374-8, PMID: 20533078

[ref95] Paço A. Duarte K. da Costa J. P. Santos P. S. M. Pereira R. Pereira M. E.. (2017). Biodegradation of polyethylene microplastics by the marine fungus *Zalerion maritimum*. Sci. Total Environ. 586, 10–15. doi: 10.1016/j.scitotenv.2017.02.017, PMID: 28199874

[ref96] Palande A. S. Kulkarni S. V. León-Ramirez C. Campos-Góngora E. Ruiz-Herrera J. Deshpande M. V. (2014). Dimorphism and hydrocarbon metabolism in *Yarrowia lipolytica* var. indica. Arch. Microbiol. 196, 545–556. doi: 10.1007/s00203-014-0990-2, PMID: 24842274

[ref97] Pang K.-L. Overy D. P. Jones E. B. G. Calado M. L. Burgaud G. Walker A. K.. (2016). ‘Marine fungi’ and ‘marine-derived fungi’ in natural product chemistry research: toward a new consensual definition. Fungal Biol. Rev. 30, 163–175. doi: 10.1016/j.fbr.2016.08.001

[ref98] Passarini M. R. Z. Rodrigues M. V. N. da Silva M. Sette L. D. (2011). Marine-derived filamentous fungi and their potential application for polycyclic aromatic hydrocarbon bioremediation. Mar. Pollut. Bull. 62, 364–370. doi: 10.1016/j.marpolbul.2010.10.003, PMID: 21040933

[ref99] Pramila R. (2011). Biodegradation of low density polyethylene (LDPE) by fungi isolated from marine water– a SEM analysis. African J. Microbiol. Res. 5, 5013–5018. doi: 10.5897/AJMR11.670

[ref100] Premnath N. Mohanrasu K. Guru Raj Rao R. Dinesh G. H. Prakash G. S. Ananthi V.. (2021). A crucial review on polycyclic aromatic hydrocarbons - environmental occurrence and strategies for microbial degradation. Chemosphere 280:130608. doi: 10.1016/J.CHEMOSPHERE.2021.130608, PMID: 33962296

[ref101] Raghukumar C. (2000). Fungi from marine habitats: an application in bioremediation. Mycol. Res. 104, 1222–1226. doi: 10.1017/S095375620000294X

[ref102] Raghukumar C. D’Souza T. M. Thorn R. G. Reddy C. A. (1999). Lignin-modifying enzymes of *Flavodon flavus*, a basidiomycete isolated from a coastal marine environment. Appl. Environ. Microbiol. 65, 2103–2111. doi: 10.1128/AEM.65.5.2103-2111.1999, PMID: 10224007 PMC91304

[ref103] Raghukumar C. D’Souza-Ticlo D. Verma A. (2008). Treatment of colored effluents with lignin-degrading enzymes: an emerging role of marine-derived fungi. Crit. Rev. Microbiol. 34, 189–206. doi: 10.1080/10408410802526044, PMID: 19003603

[ref104] Raghukumar C. Shailaja M. S. Parameswaran P. S. Singh S. K. (2006). Removal of polycyclic aromatic hydrocarbons from aqueous media by the marine fungus NIOCC # 312: involvement of lignin-degrading enzymes and exopolysaccharides. Indian J. Mar. Sci. 35, 373–379.

[ref105] Ridley C. P. Khosla C. (2009). “Polyketides,” in Encyclopedia of microbiology ed. Schaechter M. (Netherlands: Elsevier Inc), 472–481.

[ref106] Rocha L. C. Ferreira H. V. Luiz R. F. Sette L. D. Porto A. L. M. (2012a). Stereoselective bioreduction of 1-(4-methoxyphenyl)ethanone by whole cells of marine-derived fungi. Mar. Biotechnol. 14, 358–362. doi: 10.1007/s10126-011-9419-y, PMID: 22160343

[ref107] Rocha L. C. Ferreira H. V. Pimenta E. F. Berlinck R. G. S. Rezende M. O. O. Landgraf M. D.. (2010). Biotransformation of *α*-bromoacetophenones by the marine fungus *aspergillus sydowii*. Mar. Biotechnol. 12, 552–557. doi: 10.1007/s10126-009-9241-y, PMID: 19941024

[ref108] Rocha L. C. Ferreira H. V. Pimenta E. F. Berlinck R. G. S. Seleghim M. H. R. Javaroti D. C. D.. (2009). Bioreduction of *α*-chloroacetophenone by whole cells of marine fungi. Biotechnol. Lett. 31, 1559–1563. doi: 10.1007/s10529-009-0037-y, PMID: 19495566

[ref109] Rocha L. C. Lopes A. Souza D. Pereira U. Filho R. Paulo S.. (2012b). Enzymatic immobilization of marine fungi on silica gel, silica xerogel and chitosan for biocatalytic reduction of ketones. Journal Mol. Catal. B, Enzym 84, 160–165. doi: 10.1016/j.molcatb.2012.05.025

[ref110] Rocha L. C. Luiz R. F. Rosset I. G. Raminelli C. Seleghim M. H. R. Sette L. D.. (2012c). Bioconversion of iodoacetophenones by marine fungi. Mar. Biotechnol. 14, 396–401. doi: 10.1007/s10126-012-9463-2, PMID: 22653656

[ref111] Rocha L. C. Seleghim M. H. R. Comasseto J. V. Sette L. D. Porto A. L. M. (2015). Stereoselective bioreduction of *α*-azido ketones by whole cells of marine-derived fungi. Mar. Biotechnol. 17, 736–742. doi: 10.1007/s10126-015-9644-x, PMID: 26272428

[ref112] Rodrigues G. N. Alvarenga N. Vacondio B. de Vasconcellos S. P. Passarini M. R. Z. Seleghim M. H. R.. (2016). Biotransformation of methyl parathion by marine-derived fungi isolated from ascidian *Didemnum ligulum*. Biocatal. Agric. Biotechnol. 7, 24–30. doi: 10.1016/j.bcab.2016.05.001

[ref113] Saleem M. Ali M. S. Hussain S. Jabbar A. Ashraf M. Lee Y. S. (2007). Marine natural products of fungal origin. Nat. Prod. Rep. 24, 1142–1152. doi: 10.1039/b607254m 17898901

[ref114] San-Martín A. Rovirosa J. Astudillo L. Sepulveda B. Ruiz D. San-Martin C.. (2008). Biotransformation of the marine sesquiterpene pacifenol by a facultative marine fungus. Nat. Prod. Res. 22, 1627–1632. doi: 10.1080/14786410701869440, PMID: 19085420

[ref115] Serra I. Capusoni C. Molinari F. Musso L. Pellegrino L. Compagno C. (2019). Marine microorganisms for biocatalysis: selective hydrolysis of nitriles with a salt-resistant strain of *Meyerozyma guilliermondii*. Mar. Biotechnol. 21, 229–239. doi: 10.1007/s10126-019-09875-0, PMID: 30684102

[ref116] Serra I. Guidi B. Burgaud G. Contente M. L. Ferraboschi P. Pinto A.. (2016). Seawater-based biocatalytic strategy: stereoselective reductions of ketones with marine yeasts. ChemCatChem 8, 3254–3260. doi: 10.1002/cctc.201600947

[ref117] Shang Z. Salim A. A. Capon R. J. (2017). Chaunopyran A: co-cultivation of marine mollusk-derived fungi activates a rare class of 2-alkenyl-tetrahydropyran. J. Nat. Prod. 80, 1167–1172. doi: 10.1021/acs.jnatprod.7b00144, PMID: 28383912

[ref118] Shang Z. Salim A. A. Khalil Z. Bernhardt P. V. Capon R. J. (2016). Fungal biotransformation of tetracycline antibiotics. J. Org. Chem. 81, 6186–6194. doi: 10.1021/acs.joc.6b01272 27419475

[ref119] Soares P. R. S. Birolli W. G. Ferreira I. M. Porto A. L. M. (2021). Biodegradation pathway of the organophosphate pesticides chlorpyrifos, methyl parathion and profenofos by the marine-derived fungus *aspergillus sydowii* CBMAI 935 and its potential for methylation reactions of phenolic compounds. Mar. Pollut. Bull. 166:112185. doi: 10.1016/j.marpolbul.2021.112185, PMID: 33640600

[ref120] Sultan S. Noor M. Anouar E. Shah S. Salim F. Rahim R.. (2014). Structure and absolute configuration of 20*β*-hydroxyprednisolone, a biotransformed product of predinisolone by the marine endophytic fungus *Penicilium lapidosum*. Molecules 19, 13775–13787. doi: 10.3390/molecules190913775, PMID: 25255760 PMC6271985

[ref121] Thapa P. Upadhyay S. P. Suo W. Z. Singh V. Gurung P. Lee E. S.. (2021). Chalcone and its analogs: therapeutic and diagnostic applications in Alzheimer’s disease. Bioorg. Chem. 108:104681. doi: 10.1016/j.bioorg.2021.104681, PMID: 33571811 PMC7928223

[ref122] Torres J. M. O. Cardenas C. V. Moron L. S. Guzman A. P. A. dela Cruz T. E. E. (2011). Dye decolorization activities of marine-derived fungi isolated from Manila Bay and Calatagan Bay, Philippines. Philipp. J. Sci. 140, 133–143.

[ref123] Trincone A. (2011). Marine biocatalysts: enzymatic features and applications. Mar. Drugs 9, 478–499. doi: 10.3390/md9040478, PMID: 21731544 PMC3124967

[ref124] Vacondio B. Birolli W. G. Ferreira I. M. Seleghim M. H. R. Gonçalves S. de Vasconcellos S. P.. (2015a). Biodegradation of pentachlorophenol by marine-derived fungus *Trichoderma harzianum* CBMAI 1677 isolated from ascidian *Didemnun ligulum*. Biocatal. Agric. Biotechnol. 4, 266–275. doi: 10.1016/j.bcab.2015.03.005

[ref125] Vacondio B. Birolli W. G. Seleghim M. H. R. Gonçalves S. de Vasconcellos S. P. Porto A. L. M. (2015b). “Screening of marine-derived fungi isolated from the sponge *Didemnun ligulum* for biodegradation of pentachlorophenol” in Advances in bioremediation of wastewater and polluted soil. ed. Shiomi N. (UK: IntechOpen), 194–225.

[ref126] Vala A. K. Sachaniya B. Dave B. P. (2018). “Marine-derived fungi: promising candidates for enhanced bioremediation” in Approaches in bioremediation. eds. Prasad R. Aranda E. (Cham: Springer), 281–300.

[ref127] Vasconcelos M. R. S. Vieira G. A. L. Otero I. V. R. Bonugli-Santos R. C. Rodrigues M. V. N. Rehder V. L. G.. (2019). Pyrene degradation by marine-derived ascomycete: process optimization, toxicity, and metabolic analyses. Environ. Sci. Pollut. Res. 26, 12412–12424. doi: 10.1007/s11356-019-04518-2, PMID: 30847811

[ref128] Vatsal A. Zinjarde S. S. Kumar A. R. (2015). *Yarrowia lipolytica* NCIM 3589, a tropical marine yeast, degrades bromoalkanes by an initial hydrolytic dehalogenation step. Biodegradation 26, 127–138. doi: 10.1007/s10532-015-9721-x, PMID: 25708590

[ref129] Vieira G. A. L. Magrini M. J. Bonugli-Santos R. C. Rodrigues M. V. N. Sette L. D. (2018). Polycyclic aromatic hydrocarbons degradation by marine-derived basidiomycetes: optimization of the degradation process. Brazilian J. Microbiol. 49, 749–756. doi: 10.1016/j.bjm.2018.04.007, PMID: 29805073 PMC6175740

[ref130] Wang H. Ni J. Cao X. Ying G. (2010). Study on biotransformation of natural coumarin by marine fungus *Mucor* sp. MNP801 and volatile composition. Zhongguo Haiyang Yaowu 29, 6–9.

[ref131] Wu Y. R. He T. T. Lun J. S. Maskaoui K. Huang T. W. Hu Z. (2009). Removal of benzo[a]pyrene by a fungus *aspergillus* sp. BAP14. *World J*. Microbiol. Biotechnol. 25, 1395–1401. doi: 10.1007/s11274-009-0026-2

[ref132] Wu J.-S. Meng Q.-Y. Zhang Y.-H. Shi X.-H. Fu X.-M. Zhang P.. (2020). Annular oxygenation and rearrangement products of cryptotanshinone by biotransformation with marine-derived fungi *Cochliobolus lunatus* and *aspergillus terreus*. Bioorg. Chem. 103:104192. doi: 10.1016/j.bioorg.2020.104192, PMID: 32889382

[ref133] Yan W. Zhao S. S. Ye Y. H. Zhang Y. Y. Zhang Y. Xu J. Y.. (2019). Generation of indoles with agrochemical significance through biotransformation by *Chaetomium globosum*. J. Nat. Prod. 82, 2132–2137. doi: 10.1021/acs.jnatprod.8b01101, PMID: 31329433

[ref134] Yarden O. (2014). Fungal association with sessile marine invertebrates. Front. Microbiol. 5, 1–6. doi: 10.3389/fmicb.2014.00228, PMID: 24860565 PMC4030187

[ref135] Yun K. Kondempudi C. M. Choi H. D. Kang J. S. Son B. W. (2011). Microbial mannosidation of bioactive chlorogentisyl alcohol by the marine-derived fungus *Chrysosporium synchronum*. Chem. Pharm. Bull. 59, 499–501. doi: 10.1248/cpb.59.499, PMID: 21467683

[ref136] Yun K. Kondempudi C. M. Leutou A. S. Son B. W. (2015). New production of a monoterpene glycoside, 1-*O*-(*α*-d-mannopyranosyl) geraniol, by the marine-derived fungus *Thielavia hyalocarpa*. Bull. Kor. Chem. Soc. 36, 2391–2393. doi: 10.1002/bkcs.10451

[ref137] Zhang M. Z. Chen Q. Yang G. F. (2015). A review on recent developments of indole-containing antiviral agents. Eur. J. Med. Chem. 89, 421–441. doi: 10.1016/j.ejmech.2014.10.065, PMID: 25462257 PMC7115707

[ref138] Zinjarde S. S. Pant A. Deshpande M. V. (1998). Dimorphic transition in *Yarrowia lipolytica* isolated from oil-polluted sea water. Mycol. Res. 102, 553–558. doi: 10.1017/S0953756297005418

